# Effects of supplemental protein in older people: an overview of meta-analyses

**DOI:** 10.1093/ageing/afaf351

**Published:** 2025-12-12

**Authors:** Akanu Abass Obasi, Adam Lee Gordon, Kenneth Smith, Jemima T Collins, Beth E Phillips, Abdullah G Alqarni, Ying Sun, Tahir Masud, John R F Gladman

**Affiliations:** NIHR Nottingham Biomedical Research Centre (BRC), Nottingham, UK; Academic Unit of Injury, Recovery and Inflammation Sciences, School of Medicine, University of Nottingham, Nottingham, UK; Wolfson Institute of Population Health, Queen Mary University of London, London, UK; Academic Centre for Healthy Ageing, Barts Health NHS Trust, London, UK; NIHR Nottingham Biomedical Research Centre (BRC), Nottingham, UK; Academic Unit of Injury, Recovery and Inflammation Sciences, School of Medicine, University of Nottingham, Nottingham, UK; NIHR Nottingham Biomedical Research Centre (BRC), Nottingham, UK; Academic Unit of Injury, Recovery and Inflammation Sciences, School of Medicine, University of Nottingham, Nottingham, UK; NIHR Nottingham Biomedical Research Centre (BRC), Nottingham, UK; Academic Unit of Injury, Recovery and Inflammation Sciences, School of Medicine, University of Nottingham, Nottingham, UK; NIHR Nottingham Biomedical Research Centre (BRC), Nottingham, UK; Academic Unit of Injury, Recovery and Inflammation Sciences, School of Medicine, University of Nottingham, Nottingham, UK; NIHR Nottingham Biomedical Research Centre (BRC), Nottingham, UK; Academic Unit of Injury, Recovery and Inflammation Sciences, School of Medicine, University of Nottingham, Nottingham, UK; NIHR Nottingham Biomedical Research Centre (BRC), Nottingham, UK; Academic Unit of Injury, Recovery and Inflammation Sciences, School of Medicine, University of Nottingham, Nottingham, UK; Nottingham University Hospitals NHS Trust, Nottingham, UK; NIHR Nottingham Biomedical Research Centre (BRC), Nottingham, UK; Academic Unit of Injury, Recovery and Inflammation Sciences, School of Medicine, University of Nottingham, Nottingham, UK

**Keywords:** protein supplementation, community-dwelling, malnutrition, hospitalised, systematic review, older adults

## Abstract

**Background:**

Benefits of supplemental protein in older people in different health states and settings are uncertain. This review aimed to determine the effects of supplemental protein on health outcomes in older people.

**Methods:**

An overview of reviews. Cochrane Library, MEDLINE (Ovid), EMBASE (Ovid), Cumulative Index to Nursing and Allied Health Literature (CINAHL) (EBSCOhost) and Google Scholar were searched from January 1990 to August 2024. Systematic reviews with meta-analyses reporting protein intake’s effect on health outcomes were included. Methodological quality was assessed using the Assessing Methodological Quality of Systematic Reviews 2 critical appraisal tool. Inconsistency between results from different reviews was explored by examining effects by population type [healthy people, living with long-term conditions (LTCs), hospital inpatients] and whether supplementation was given with or without concomitant exercise.

**Results:**

Thirty-three reviews with meta-analyses collating data from 441 unique studies were included. There was no increase in muscle mass, strength or physical performance from protein supplementation in older people in general, nor in healthy older people. There was medium-certainty evidence of at least small increases in muscle mass and strength in older people with LTCs, and the benefits of protein supplementation were more certain with concomitant exercise. Evidence suggests that protein supplementation in hospital patients with hip fractures reduced the number of medical complications.

**Conclusion:**

Protein supplementation is effective for improving muscle mass and strength in older people with LTCs and medical complications in older hospitalised patients with hip fractures. The evidence does not support its routine use in other groups of healthy older people.

## Key Points

- Effects of protein supplementation depend upon who is supplemented.
- Older people with long-term conditions are likely to benefit from protein supplementation (increased mass and strength) with exercise.
- Healthy older people seem not to benefit from protein supplementation with or without concomitant exercise.
- Older hospitalised hip fracture patients benefit from protein supplementation in terms of medical complications reduction.

## Introduction

Dietary protein is vital for optimal muscle function, regulating body processes, transporting materials, fluid balance, immunity, respiration and programmed cell death [1]. The benefits of protein supplementation may differ between, healthy older people [2]- traditionally-defined as the absence of chronic diseases [3]; although defined by the World Health Organisation differently [4], those living with long-term conditions (LTCs)- ‘health problems that need ongoing management over a period of months/years/or decades, and cannot currently be cured but can be controlled with medication and/or other therapies’ [5]: where inactivity and ‘anabolic resistance’ impair protein metabolism [6]; and hospitalised people where inactivity and inflammatory burden are common [7]. Effects of protein supplementation are also dependent upon the amount of physical activity accompanying supplementation [8].

Ageing is associated with adverse outcomes including sarcopenia (loss of muscle mass and strength) [9] and frailty (vulnerability to poor resolution of homeostasis after stressors) [10]. Physical activity and nutrition are each factors associated with the development of both sarcopenia and frailty [11, 12], explaining why interventions promoting activity and optimising nutrition have been tested to mitigate sarcopenia and frailty.

This overview of reviews aimed at closing a gap in knowledge on the effects of supplemental protein on health outcomes in older people.

## Methods

Following Preferred Reporting Items for Overviews of Reviews and Cochrane Handbook guidance [13, 14], and after registration with PROSPERO (CRD42023412918), the following research questions were explored:

- Do older people benefit from protein supplementation?
- Does the benefit of protein supplementation depend on who is supplemented?
- Do older people benefit from protein supplementation when given with or without exercise?

Records were screened stepwise against eligibility criteria (Supplementary Appendix SS1). Using search algorithms developed with a clinical librarian the Cochrane Library, MEDLINE (Ovid), Embase (Ovid), CINAHL (EBSCOhost) and Google Scholar were searched from January 1990 to 6 August 2024.

**Figure 1:**
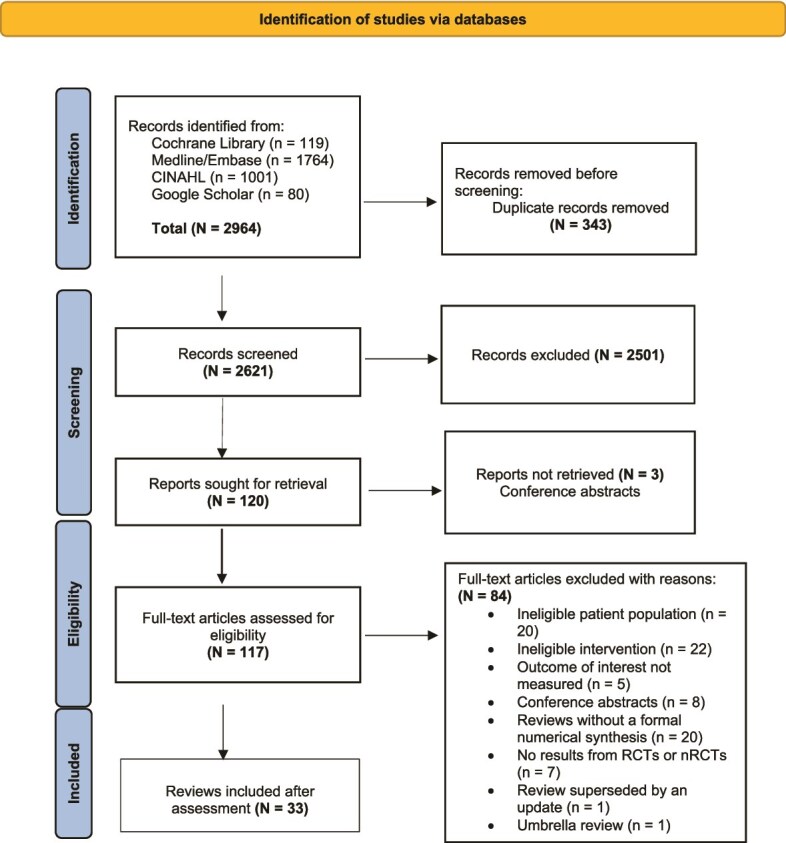
PRISMA diagram. *n*, number; nRCTs, non-randomised control trials.

MeSH terms and free text were combined. Our search included meta-analyses and systematic reviews (SRs) of randomised controlled trials (RCTs) with no language restrictions. Papers not in English were translated (Supplementary Appendix SS2 for full search strategy).

Three authors (A.A.O., Y.S., A.G.A.) independently screened records for eligibility and then assessed full texts of eligible articles. Three authors (A.A.O., Y.S., J.R.F.G.) independently extracted data using full duplicate data entry. Disagreements were resolved by discussion.

Data extracted included: paper title; author; publication year; first author country; language; number and name of electronic databases searched; languages included; search dates; PICO (patient, intervention, comparison and outcome); study designs; studies with meta-analysis; timeframe of intervention; meta-analysis results [effect sizes, confidence intervals (CIs), *P*-values, heterogeneity]; and level of certainty for each outcome. No assumption was made on unavailable or unclear data. Three authors (A.A.O., Y.S., J.R.F.G.) independently assessed risk of bias using the Assessing Methodological Quality of Systematic Reviews 2 (AMSTAR-2) tool [15], resolving differences through discussion (Supplementary Appendix SS3). Reviews were rated by number of weaknesses in ‘critical’ and ‘non-critical’ checklist items [15] (Supplementary Appendix SS4). The Grading of Recommendations Assessment, Development and Evaluation system [16] was used by two independent reviewers (A.A.O. and J.R.F.G.) to rate certainty of extracted results, classified as high, moderate, low or very low certainty (Supplementary Appendix SS6).

Synthesis was narrative, using only meta-analysis results or network meta-analysis results. Four population groups were selected: healthy older people, those with LTCs such as sarcopenia or frailty, hospitalised patients and mixed populations (healthy and LTCs). For each of these populations, meta-analysis results for studies where protein supplementation was given without concomitant exercise, with concomitant exercise, and where studies with and without concomitant exercise were combined were selected. Where meta-analysis results for an outcome of interest was given for more than one time point, the result including the largest number of studies was selected. Where more than one meta-analysis result for an outcome of interest was given, only one result was selected using the following hierarchies. For mass: appendicular skeletal muscle mass index > appendicular skeletal muscle mass > muscle mass > lean body mass. For strength: knee extension > leg press > handgrip. For performance: short physical performance battery > chair rise test > timed up and go > gait speed. Where a meta-analysis result was presented as mean difference (MD) or weighted mean difference (WMD) and the standard deviation of the constituent groups were available, standardised mean difference (SMD) was calculated. SMD results were plotted in forest plots and MD and WMD tabulated.

Consistency of all results extracted for effects on muscle mass, muscle strength and physical performance was examined. Where results were inconsistent, their consistency by participant type (healthy older people, older people with LTCs and hospitalised older patients) was examined. If these results remained inconsistent, results were further subdivided into those when protein supplementation was given without or with concomitant exercise.

Initial consistency of results was determined thus: if there was an overlap in the 95% CI limits of SMD results, and if there were no statistically significant MD/WMD results that were incompatible with this overlap. If there was no such overlap, it was deemed that there was consistent evidence of an effect if limits of 95% CIs fell within the same effect zone, and if MD/WMD results were not incompatible with such an effect. The effect zones were defined as follows: no/negligible effect: −0.19 to 0.19, small effect: 0.2 to 0.59, medium effect: 0.6 to 0.79, large effect ≥0.8) [17]. Findings were summarised as either consistent or inconsistent for each outcome domain and each population group and subgroup.

Certainty ratings were then applied to these findings. High certainty findings were consistent and drawn from at least one high-quality result. Medium certainty findings were consistent and drawn from more than one non-high-quality result. Low certainty results were inconsistent findings, or findings drawn from only one non-high-quality result. Findings that were imprecise (i.e. 95% CIs crossing the boundary between a small effect or no/negligible effect) were downgraded to the next lower rating.

## Results

### Characteristics of included systematic reviews/meta-analysis

Included SRs were published from 2014 to 2024. They reviewed a total of 441 unique studies from which 3–78 studies were cited per article, all including results for men and women. One-hundred and thirty-five results were extracted, of which 58 assessed muscle mass, 48 muscle strength, 27 physical performances and 1 for medical complications and mortality. Reviews reported protein doses of 0.6–125 g/day with 1 to 108-week intervention durations. Thirty-one reviews had meta-analyses restricted to RCTs [18–48] and two had meta-analyses including RCTs and quasi-CTs [49, 50] (Supplementary Appendix SS5).

Risk of bias for the 33 reviews are summarised in Supplementary Appendix SS3. One was high-quality [50], four were low-quality [24, 39, 42, 43] and twenty-eight critically low-quality [18–23, 25–27, 29–38, 40, 41, 44–49].

Certainty of the 135 results assessed are summarised in Supplementary Appendix SS6. Included reviews provided very low (*n* = 25), low (*n* = 34) or moderate-certainty (*n* = 76) evidence.

### Effects of interventions on muscle mass

**Figure 2:**
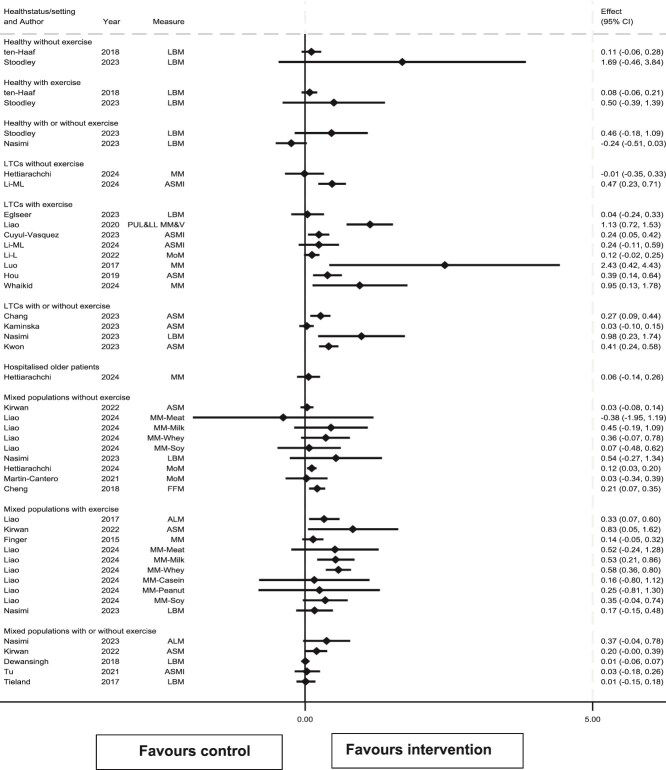
Forest plot of review results for the effect of protein supplementation upon muscle mass, presented by participant type and whether protein supplementation was given in the presence of concomitant exercise. ALM, appendicular lean mass; ASM, appendicular skeletal muscle mass; ASMI, appendicular skeletal muscle mass index; LBM, Lean body mass; CSA, cross-sectional area; FFM, fat-free mass; LBM, lean body mass; MM, muscle mass; MoM, mixture of measures; PUL&LL MM&V, Pooled upper limb and lower limb muscle mass and volume.

Of 58 meta-analysis results for muscle mass across all populations and exercise categories, 45 reported SMD (Figure 2) and 13 either MD or WMD (Table 1). Results were generally in favour of protein supplementation: 54/58 results showed effects >0 (42/45 SMD results and 12/13 MD/WMD). However, only 18/58 results (14/45 SMD and 4/13 MD/WMD) showed these benefits to be statistically significant (lower 95% CI > 0.0 or *P* < .05) and only 7/45 SMD results excluded no or negligible benefit (SMD ≥ 0.20).

**Table 1:** WMD, MD and RR results for included studies.^a^

Review	Population and measure	WMD/MD/RR	95% confidence interval
	**Muscle mass**		
	**Healthy older people, without concomitant exercise**		
Kirwan 2022 [32]	Appendicular lean mass	WMD 0.26	−0.42, 0.95
	**Healthy older people, with concomitant exercise**		
Hidayat 2018 [48]	Fat-free muscle	MD 0.32	−0.16, 0.79
Kirwan 2022 [32]	Appendicular lean mass	WMD −0.08	−0.96, 0.80
	**Healthy older people, with and without concomitant exercise**		
None	N/A	N/A	N/A
	**Older people with long-term conditions, without concomitant exercise**		
Kirwan 2022 [32]	Appendicular lean mass	WMD 0.30	−0.13, 0.20
	**Older people with long-term conditions, with concomitant exercise**		
Kirwan 2022 [32]	Appendicular lean mass	WMD **0.88**	0.51, 1.25
Hidayat 2018 [48]	Fat-free mass	MD **1.62**	0.92, 2.28
	**Older people with long-term conditions, with and without concomitant exercise**		
Kirwan 2022 [32]	Lean body mass	WMD 0.50	−0.17, 1.17
Oktaviana 2020 [42]	Lean body mass	MD 1.17	−1.97, 4.30
Ren 2023 [46]	Appendicular skeletal muscle mass	MD 0.08	−0.01, 0.16
	**Mixed older populations, without exercise**		
Beaudart 2018 [43]	Appendicular lean mass	MD 0.06	−0.27, 0.15
	**Mixed older populations, with exercise**		
Hidayat 2018 [48]	Fat-free mass	MD **0.74**	0.30, 1.17
	**Mixed older populations, with and without concomitant exercise**		
Ren 2023 [46]	Appendicular skeletal muscle mass	MD **0.82**	0.55, 1.09
Xu 2014 [47]	Lean body mass	MD 0.34	−0.42, 1.10
	**Strength**		
	**Healthy older people, without concomitant exercise**		
None	N/A	N/A	N/A
	**Healthy older people, with concomitant exercise**		
None	N/A	N/A	N/A
	**Healthy older people, with and without concomitant exercise**		
None	N/A	N/A	N/A
	**Older people with long-term conditions, without concomitant exercise**		
Kirwan 2022 [32]	Handgrip	WMD 0.0	−0.41, 0.41
	**Older people with long-term conditions, with concomitant exercise**		
Kirwan 2022 [32]	Handgrip	WMD **2.06**	0.66, 3.47
	**Older people with long-term conditions, with and without concomitant exercise**		
None	N/A	N/A	N/A
	**Mixed older populations, without exercise**		
None	N/A	N/A	N/A
	**Mixed older populations, with concomitant exercise**		
None	N/A	N/A	N/A
	**Mixed older populations, with and without concomitant exercise**		
Xu 2014 [47]	Leg extension	MD 2.28	−1.73, 6.29
Kirwan 2022 [32]	Knee extension	WMD 1.88	−0.60, 4.35
Ren 2023 [46]	Handgrip	MD **0.43**	0.18, 0.68
	**Physical performance**		
	**Healthy older people, without concomitant exercise**		
None	N/A	N/A	N/A
	**Healthy older people, with concomitant exercise**		
None	N/A	N/A	N/A
	**Healthy older people, with and without concomitant exercise**		
None	N/A	N/A	N/A
	**Older people with long-term conditions, without concomitant exercise**		
None	N/A	N/A	N/A
	**Older people with long term conditions, with concomitant exercise**		
None	N/A	N/A	N/A
	**Older people with long-term conditions, with and without concomitant exercise**		
Oktaviana 2020 [42]	Short Physical Performance Battery	MD 0.61	−0.02, 1.23
	**Mixed older populations, without concomitant exercise**		
None	N/A	N/A	N/A
	**Mixed older populations, with concomitant exercise**		
None	N/A	N/A	N/A
	**Mixed older populations, with and without concomitant exercise**		
Ren 2023 [46]	Timed Up and Go test	MD 0.21	0.15, 0.26
	**Older people in hospital, without concomitant exercise**		
Avenell 2016 [50]	Mortality	RR 1.42	0.85, 2.37
Avenell 2016 [50]	Inpatient medical complications	RR **0.78**	0.65, 0.95
	**Older people in hospital, with concomitant exercise**		
None	N/A	N/A	N/A
	**Older people in hospital, with and without concomitant exercise**		
None	N/A	N/A	N/A

^a^Bold text denotes statistically significant results. N/A rows are where results are reported as SMD or not used to avoid duplications or not available for analysis.

SMD results were inconsistent, with no overlap between 95% CIs, and no overlap in effect zones subtended by the 95% CIs. Seven from 45 showed results where the lower 95% CI limit was ≥0.20, indicating a benefit of a magnitude that excluded no or negligible benefit, 5/45 results had an upper 95% CI limit <0.20, thus excluding anything other than no or negligible benefit.

To examine this inconsistency, effect sizes were explored by study population. Nine results were drawn solely from healthy populations (6 SMD and 3 MD/WMD results), and 20 from those with LTCs (14 SMD and 6 MD/WMD results). There was only one muscle mass result drawn from hospitalised patients: this showed no or negligible effect but did not exclude a small benefit (SMD 0.06, 95% CI −0.14 to 0.26) [21].

Although effect of protein supplementation was generally positive in healthy populations (7/9 results, 5/6 SMD and 2/3 MD/WMD, were > 0.0), none of these results were statistically significant (lower 95% CI > 0.0 or *P* < .05). There was consistency in the results in that overlap in 95% CI of SMD results was from −0.03 to 0.06 indicating no or negligible benefit.

Effect of protein supplementation in populations with LTCs was also generally positive (18/20 results: 13/14 SMD and 5/6 MD/WMD, were >0.0), and 11/20 results (9/14 SMD and 2/6 MD/WMD) showed statistically significant benefit. SMD results were inconsistent in that there was no overlap in 95% CIs, although there was near consistency in terms of effect zones subtended by their 95% CIs: 13/14 results were compatible with a benefit that excluded no or negligible effect. The sole result that excluded a small benefit (Kaminska, SMD 0.03, 95% CI −0.10 to 0.15) [30] was drawn from 10 studies restricted to whey protein, combined studies with and without concomitant exercise, and was rated as critically low-quality (Supplementary Appendix SS3).

Given that there was potential inconsistency between results in the LTCs group, a further sub-analysis of results from studies of protein supplementation with or without concomitant exercise was conducted. There were 3 results in populations with LTCs where protein supplementation was given without concomitant exercise (2 SMD, 1 MD), and 10 results when exercise was given concomitantly with protein supplementation (8 SMD, 2 MD). Two of the three results where exercise was not given showed benefit, but none were statistically significant. However, there was an overlap in the 95% CIs of the two SMD results (0.23 to 0.33) indicating a consistent small benefit. Although there was no overlap in the 95% CIs of the eight SMD results where concomitant exercise was given, all results were compatible with at least a small benefit (upper 95% CI limit ≥0.20).

In summary, there was medium certainty evidence that protein supplementation in healthy older people has no or negligible effect on muscle mass. In older people with LTCs, there was medium certainty evidence of an increase in muscle mass due to protein supplementation—a small increase when given without concomitant exercise, and at least a small increase when given with concomitant exercise. There was low certainty evidence of no or negligible effect of supplementation on muscle mass in hospitalised patients, with no evidence to estimate the effect of concomitant exercise in this population.

### Effects of interventions on muscle strength

**Figure 3:**
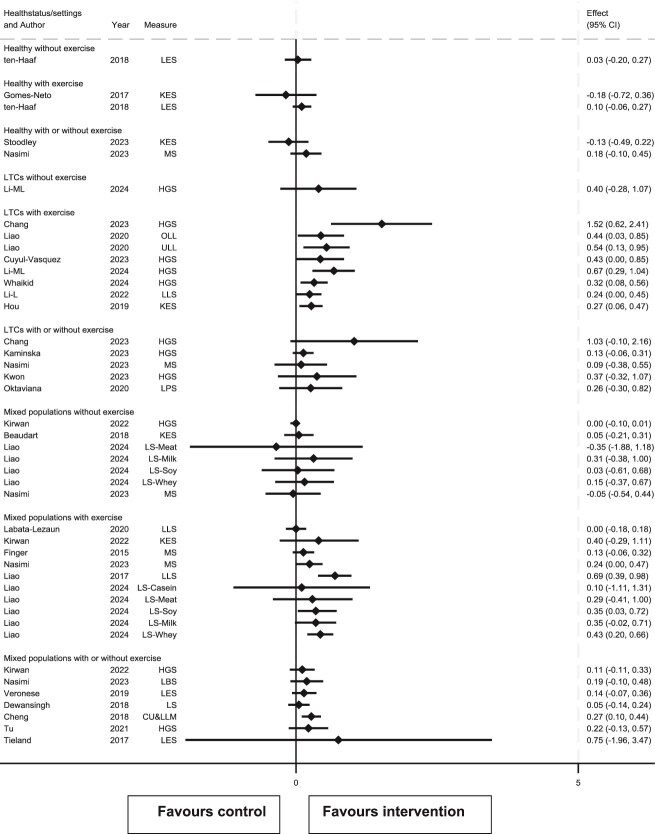
Forest plot of review results for the effect of protein supplementation upon muscle strength, presented by participant type and whether protein supplementation was given in the presence of concomitant exercise. CU&LLM, combined upper and lower limb measures; HGS, handgrip strength; KES, knee extensor strength; LBS, lower body strength; LES, lower extremity strength; LLS, lower limb strength; LPS, leg press strength; LS, leg strength; MIS, maximal isometric strength; MS, muscle strength; OLL, operated lower limb; ULL, unoperated lower limb; UBS, upper body strength.

Of 48 meta-analysis results for muscle strength across all populations and exercise categories, 43 reported SMD (Figure 3) and 5 either MD or WMD (Table 1). Results were generally in favour of protein supplementation: 43/48 results showed effects >0 (39/43 SMD results and 4/5 MD/WMD). However, only 11/48 results (9/43 SMD and 2/5 MD/WMD) were statistically significant (lower 95% CI >0 or *P* < .05) and only 4/43 SMD results excluded no or negligible benefit (lower 95% CI, SMD ≥ 0.20).

SMD results were inconsistent: there was no overlap between 95% CIs of SMD results, nor of the effect zones subtended by their 95% CIs.

To examine this inconsistency, effect sizes by study population were explored. Five results were drawn solely from healthy populations (5 SMD, no MD/WMD results), and 16 from those with LTCs (14 SMD and 2 MD/WMD results). There were no results from hospitalised patients.

The effect of protein supplementation on muscle strength in healthy populations was unclear: effects of 3/5 results were > 0.0. Further, none of the positive effects were statistically significant. However, there was consistency: overlap in 95% CI of SMD results was from −0.06 to 0.22 indicating no or negligible benefit but a small benefit was not confidently excluded. Findings were similar for results from studies without (overlap −0.06 to 0.23) and with concomitant exercise (overlap also −0.06 to 0.23).

Effect of protein supplementation on muscle strength in populations with LTCs was strongly positive (15/16 results, 14/14 SMD and 1/2 MD/WMD were > 0.0), with 7/16 results (6/14 and 1/2 MD/WMD) statistically significant. There was no overlap in 95% CIs or in effect zones of the 14 SMD results, but these inconsistencies arose from two results with non-overlapping CIs: Kaminska (95% CI −0.06 to 0.31) [30] and Chang (95% CI 0.62 to 2.41) [27]. However, results were consistent in that they were all compatible with at least a small benefit (upper 95% CI limits all ≥0.20).

Given this minor inconsistency in results in the LTCs group, a further sub-analysis of results from studies of protein supplementation with or without concomitant exercise was conducted. There was only one (SMD) result in populations with LTCs where protein supplementation was given without concomitant exercise (Li), which showed a small but imprecise benefit (SMD 0.40, 95% CI −0.28 to 1.07) [22]. Exercise was given concomitantly with protein supplementation in 9 results (8 SMD, 1 MD), with no overlap in 95% CIs of the 8 SMD results nor in their effect zones. However, all results were compatible with at least a small benefit (upper 95% CI limit ≥0.20).

In summary, there was low certainty evidence that protein supplementation in healthy older people has no or negligible effect on muscle strength. In older people with LTCs, there was low certainty evidence of an increase in muscle strength due to protein supplementation: there was very low certainty evidence of a small benefit in those given protein supplements without concomitant exercise but medium certainty evidence of at least a small benefit when supplemented with concomitant exercise. We found no evidence of protein supplementation effect on muscle strength in hospitalised patients.

### Effects of interventions on physical performance

**Figure 4:**
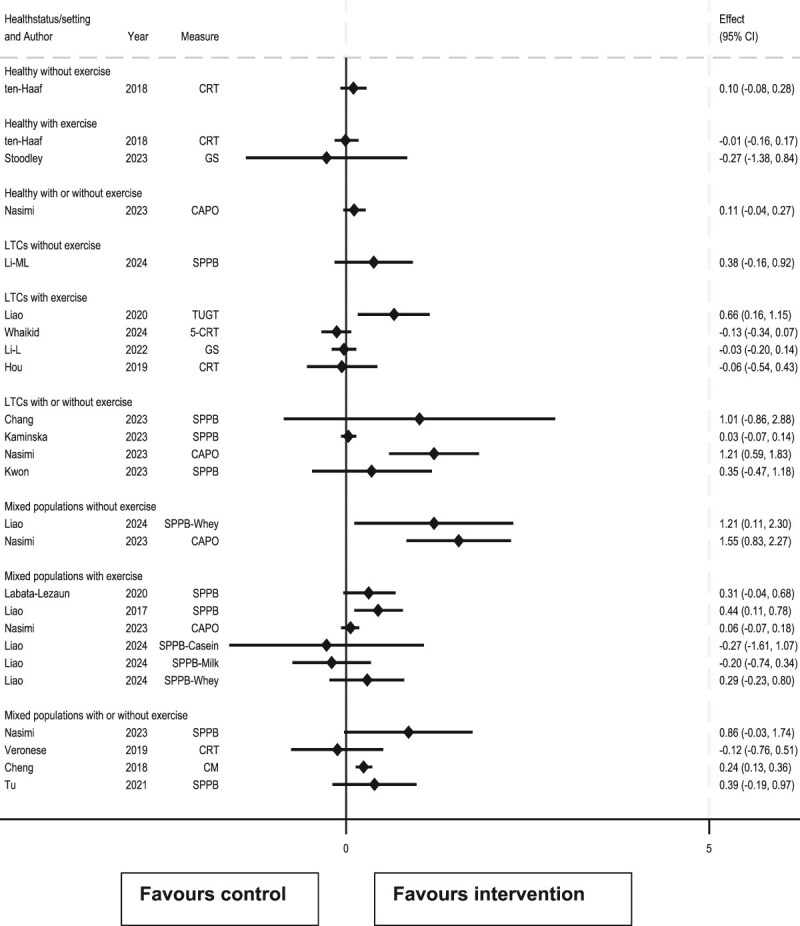
Forest plot of review results for the effect of protein supplementation upon physical performance, presented by participant type and whether protein supplementation was given in the presence of concomitant exercise. CAPO, composite of all performance outcomes; CM, combined measures; CRT, chair rise test; GS, gait speed; LTCs, long-term conditions; OPT, other physical test; SC, stair climbing; SPPB, short physical performance battery; TUGT, timed up & go test; WC, walking capacity; WS, walking speed.

Of 27 meta-analysis results for physical performance across all populations and exercise categories, 25 reported SMD (Figure 4) and 2 either MD or WMD (Table 1). Of these, 19/27 showed effects >0 (17/25 SMD results and 2/2 MD/WMD). However, only 5/27 results (5/25 SMD and 0/2 MD/WMD) were statistically significant (lower 95% CI >0 or *P* < .05) and only 2/25 SMD results excluded no or negligible benefit (lower 95% CI ≥0.20).

SMD results were inconsistent: there was no overlap between 95% CIs of SMD results, nor of the effect zones subtended by their 95% CIs.

To examine this inconsistency, effect sizes by study population were determined. Four (SMD) results were drawn solely from healthy populations, and 10 from those with LTCs (9 SMD and 1 MD/WMD results). There were no results from hospitalised patients.

The general effect of protein supplementation on physical performance in healthy populations was unclear: the effects of 2/4 results were > 0.0, neither of which were statistically significant. However, there was consistency in the results, where the overlap in 95% CI of SMD results was 0.04 to 0.17 indicating no or negligible benefit. The findings were similar when results came from studies without (overlap −0.08 to 0.26) and with concomitant exercise (overlap −0.16 to 0.17).

The effect of protein supplementation on physical performance in populations with LTCs was generally positive (7/10 results, 6/9 SMD and 1/1 MD/WMD were >0.0), but only 2/10 results (2/9 SMD and 0/1 MD/WMD) were statistically significant. There was no overlap in 95% CIs or in effect zones of the 9 SMD results, but these inconsistencies arose from the sole statistically significantly positive result (Nasimi) with 95% CI 0.59 to 1.83 [20]. The Nasimi review studied the effect of whey protein in 22 studies and was rated as critically low-quality. Excluding this, there was overlap of 95% CI of SMD results of −0.07 to 0.07 indicating no or negligible effect.

Given these minor inconsistencies in results in the LTCs group, a further sub-analysis of results from studies of protein supplementation with or without concomitant exercise was conducted. There was only one (SMD) result in populations with LTCs where protein supplementation was given without concomitant exercise (Li), showing a small but imprecise benefit (SMD 0.38, 95% CI −0.16 to 0.92) [22]. There were 5 results when exercise was given concomitantly with protein supplementation (4 SMD, 1 MD) all of which were positive, one of which was statistically significant (Nasimi), with no overlap in the 95% CIs of the SMD results nor in effect zones [20]. With the Nasimi study excluded, the remaining studies were compatible with no or negligible effect (95% CI −0.07 to 0.14).

In summary, there was medium certainty evidence that protein supplementation in healthy older people has no or negligible effect on physical performance. In older people with LTCs, there was low certainty evidence of no or negligible effect on physical performance. We found no evidence of effect of supplementation on physical performance in hospitalised patients.

### Other outcomes

Only one review (Avenell, a high quality review restricted to in-patients with hip fracture) [50], reported effects of protein supplementation on mortality and in-patient medical complications. Mortality was non-significantly increased: relative risk (RR) 1.42, 95% CI 0.85 to 2.37, yet in-patient medical complications were significantly reduced: RR 0.78, 95% CI 0.65 to 0.95 [50].

## Discussion

Overall, there was no increase in muscle mass, muscle strength or physical performance from protein supplementation in older people in general, nor in healthy older people. However, there was medium certainty evidence of at least small increases in muscle mass and muscle strength in older people with LTCs, and benefits of protein supplementation were more certain with concomitant exercise. In addition, protein supplementation in hospitalised patients (with hip fracture) reduced the number of medical complications.

That relevant review evidence was sought through systematic searching, drawing upon meta-analysis results only increases confidence in the robustness of these findings. However, although heterogeneity was explored by considering different study populations and presence of concomitant exercise, other factors which could influence effect of protein supplementation such as participants’ habitual diet, and type, dose and timing of protein supplementation and exercise were not explored. We recognise that many necessary conditions are required to mediate translation of increases in muscle mass and muscle strength into improved physical performance, such as training in physical performance rather than resistance exercise alone. One interpretation of our finding that protein supplementation did not improve physical performance is because these necessary conditions were not applied in these studies.

The findings presented herein are limited by paucity of primary evidence (leading to imprecise effect size estimates); high heterogeneity within evidence (reflecting the large variety of potential participants, supplements, and exercise regimes) and concerns about the quality of reviews included. The lack of overlap in primary studies between similar reviews may reflect limitations of our search terms and/or those deployed in the studies cited. The methodologies used here focussed on between group differences in RCTs and do not enable understanding of whether the mechanism of effect was maintenance of muscle mass in intervention groups, versus muscle loss in controls [51]; or whether there was muscle gain in intervention groups. It is likely that different mechanisms contributed in different studies and population groups.

Our finding that muscular outcomes were improved by supplementation with concomitant exercise is supported by knowledge that muscle hypertrophy requires both amino acid availability and contractile stimulus [52]. Exercise augments the effects of protein supplementation, and both healthy older people and those with LTCs can benefit [53, 54].

Our findings imply there is no justification for proposing protein supplementation for all older people or healthy older people. However, trial populations may not be fully representative of the populations from which they were drawn and so there may be apparently healthy individuals who could benefit from increased protein intake [55]. Further, only short-term (typically 1–108 weeks) effects of protein supplementation was examined and therefore longer-term increased protein intake effects on conditions such as sarcopenia could not be determined.

Older people with hip fracture are likely to be considerably undernourished [56]. The finding that protein supplementation reduces complications in this group may reflect an effect mediated by improved immune function and wound healing rather than increased muscle mass and strength [57–59]. This finding may well generalise to other groups of frail older in-patients with injurious and catabolic conditions. One implication is that overall nutritional support for older people in such states, which will include other nutrients such as calories and vitamins [51], should include a high protein component.

Our finding of benefit on muscle mass from protein supplementation with or without concomitant exercise in older people with LTCs is novel as previous overviews of reviews [60, 61] did not conduct this subgroup analysis. Guidance for the care of older people with LTCs should incorporate this finding. It is worth noting, though, that LTCs are heterogenous pathophysiologically and the effectiveness of protein supplementation may vary between conditions. Because of the limitations of the literature, further secondary analysis of existing studies is unlikely to address the relative contribution of protein supplementation in different conditions. Future primary research should focus on more detailed factors such as dose, type and timing of protein supplementation and exercise regimes, including means to implement these findings at scale.

In conclusion, healthy older adults do not benefit from protein supplementation with or without concomitant exercise. Older people with LTCs demonstrated benefit in terms of muscle mass and strength, particularly with concomitant exercise. More well-designed clinical trials of good methodological quality are warranted to understand the nuances of how best to supplement protein, which adjuncts best facilitate clinical effectiveness and how specific LTCs respond to supplementation.

## Supplementary Material

aa-25-2019-File002_afaf351
