# Supplementary material for: Effects of supplemental protein in older people: an overview of meta-analyses
Source: Age Ageing. 2025 Dec 12;54(12):afaf351. doi: 10.1093/ageing/afaf351 (PMC12700089; doi:10.1093/ageing/afaf351)
Supplement: aa-25-2019-File002_afaf351 [file aa-25-2019-file002_afaf351.docx]

**Effects of supplemental protein in older people: an overview of meta-analyses**

**SUPPLEMENTARY DATA SECTION**

**Contents List:**

**Appendix 1.** Eligibility criteria of systematic reviews (SRs) of randomised controlled trials with meta-analyses.

**Appendix 2.** Search strategy

[**Appendix 3.** Showing critical appraisal of included systematic reviews and meta-analyses using the AMSTAR 2 tool](#_Table_6._AMSTAR)

**Appendix 4.** AMSTAR-2 Critical Checklist

**Appendix 5.** Characteristics of the reviews included

**Appendix 6.** Results of the GRADE Certainty Assessment

**Appendix 7.** Showing excluded reviews with reasons

**Appendix 1.** Eligibility criteria of systematic reviews (SRs) of randomised controlled trials with meta-analyses.

| **Category** | **Inclusion criteria** | **Exclusion criteria** |
| --- | --- | --- |
| **Participant** | -SRs that focus upon or provide specific results for older people with a mean or median age of ≥60 years.  -SRs were eligible irrespective of participants’ place of residence, nutritional or illness status. |  |
| **Intervention** | -SRs that focus upon, or provide specific results for, the effect of protein intake upon the outcome, whether consumed as food or a nutritional fortification or supplement, administered via an enteral tube, or delivered parenterally.  -SRs that evaluated the impact of protein alongside exercise were also included. | -SRs that provide results only supplementation of essential amino acids or their metabolites were ineligible. |
| **Comparator** | -SRs that focus upon or provide specific results, where the comparison is a nutritional intake matched for micro- or macro-nutrients other than protein and any other experimental intervention. |  |
| **Outcomes** | -SRs that provide data on nutritional outcomes (such as body weight, or nutritional biomarkers), functional outcomes (such as muscle mass, muscle strength, mobility or gait speed, dependency), clinical outcomes (such as complications, mortality, survival), and healthcare service outcomes (such as length of stay, non-elective readmissions, costs). |  |
| **Study design** | -SRs that provided results from randomised controlled trials or well-conducted non-randomised controlled trials.  - SRs that included results only from observational (non-experimental) studies were eligible only if propensity-matched analyses, or multivariate regression analyses, taking account of patient and other intervention factors were reported. This was to avoid the confounding effects of other factors (such as other nutrients) and to reduce selection bias (such as when the amount of protein given is adjusted according to prognostic factors). | -Umbrella reviews  - Reviews without a formal numerical synthesis  -Conference abstracts |

**Appendix 2.** Search strategy

| CDSR [COCHRANE LIBRARY] Search Strategy Interface: Wiley | |
| --- | --- |
|  | **searches** |
| #1 | mesh descriptor: [dietary proteins] explode all trees |
| #2 | mesh descriptor: [dietary supplements] this term only |
| #3 | (((protein* near/3 (breakdown or casein or intake or optimal or quality or recommendations or requirements or soya or supplement* or synthesis or turnover or whey)) or "net protein balance" or "energy balance" or "metabolic adaptation")):ti,ab,kw |
| #4 | #1 or #2 or #3 |
| #5 | mesh descriptor: [aged] explode all trees |
| #6 | mesh descriptor: [geriatrics] this term only |
| #7 | ((ageing or aging or elderly or geriatric* or senior or (older near/1 (adult* or man or men or woman or women or person* or people)))):ti,ab,kw |
| #8 | ((centenarian or gerontolog* or nonagenarian or octogenarian or septuagenarian or sexagenarian)):ti,ab,kw |
| #9 | ((age* near/3 (over or older) near/2 (6# or 7# or 8# or 9#))):ti |
| #10 | ((age* near/3 (over or older) near/2 (6# or 7# or 8# or 9#))):ab |
| #11 | ((">=6# years old" or ">6# years old" or (">=7# years old" or ">7# years old") or (">=8# years old" or ">8# years old") or (">=9# years old" or ">9# years old"))):ti |
| #12 | ((">=6# years old" or ">6# years old" or (">=7# years old" or ">7# years old") or (">=8# years old" or ">8# years old") or (">=9# years old" or ">9# years old"))):ab |
| #13 | #5 or #6 or #7 or #8 or #9 or #10 or #11 or #12 |
| #14 | #4 and #13 |
| Embase 1974 to 2024 August 6, Ovid MEDLINE(R) ALL 1946 to August 6, 2024. Interface: OVID | |
| #1 | (exp dietary proteins/ or dietary supplements/) use medall |
| #2 | (diet supplementation/ or ((casein or protein* or soyabean).sh. and supplementation/)) use oemezd |
| #3 | ((protein* adj3 (breakdown or casein or intake or optimal or quality or recommendations or requirements or soya or supplement* or synthesis or turnover or whey)) or net protein balance or energy balance or metabolic adaptation).tw. |
| #4 | or/1-3 |
| #5 | (exp aged/ or geriatrics/) use medall |
| #6 | (exp aged/ or exp geriatrics/) use oemezd |
| #7 | (ageing or aging or elderly or geriatric* or senior or (older adj (adult? or m#n or wom#n or person? or people))).tw,kw. |
| #8 | (centenarian or gerontolog* or nonagenarian or octogenarian or septuagenarian or sexagenarian).tw,kw. |
| #9 | (age? adj3 (over or older) adj2 (6# or 7# or 8# or 9#)).tw. |
| #10 | (">=6# years old" or ">6# years old" or (">=7# years old" or ">7# years old") or (">=8# years old" or ">8# years old") or (">=9# years old" or ">9# years old")).tw. |
| #11 | or/5-10 |
| #12 | meta-analysis as topic/ or meta-analysis/ or exp review literature as topic/ or “systematic review”/ |
| #13 | (data extraction or selection criteria).ab. and review/ |
| #14 | exp meta analysis/ or “systematic review”/ |
| #15 | (data extraction or selection criteria).ab. and review.pt. |
| #16 | (meta analy* or metaanaly* or (systematic adj (review*1 or overview*1))).tw. |
| #17 | (bids or cancerlit or cinahl or cinhal or cochrane or embase or psychinfo or psycinfo or psychlit or psyclit or science citation index).ab. |
| #18 | (bibliograph* or hand-search* or manual search* or reference list* or relevant journals).ab. |
| #19 | (or/12-13,16-18) use medall |
| #20 | (or/14-18) use oemezd |
| #21 | or/19-20 |
| #22 | and/4,11,21 |
| #23 | (comment/ or editorial/ or letter/ or (animal/ not human/)) use medall |
| #24 | ((editorial or letter).pt. or (animal/ not human/)) use oemezd |
| #25 | 22 not (or/23-24) |
| #26 | 25 |
| #27 | limit 26 to yr="1990 -current" |
| CINAHL literature search. Interface: EBSCO | |
| s21 | s17 not s20 |
| s20 | s18 or s19 |
| s19 | (mh "animals") |
| s18 | pt commentary or letter or editorial |
| s17 | s4 and s12 and s16 |
| s16 | s13 or s14 or s15 |
| s15 | tx meta analys* or metaanaly* or (systematic n1 (review or overview)) |
| s14 | (mh "meta analysis") |
| s13 | (mh "literature review+") or (mh “systematic review”) |
| s12 | s5 or s6 or s7 or s8 or s9 or s10 or s11 |
| s11 | ti (elderly or geriatric* or senior) or ab (elderly or geriatric* or senior) or su (elderly or geriatric* or senior) |
| s10 | ti (older n1 (adult# or m?n or wom?n or person# or people)) or ab (older n1 (adult# or m?n or wom?n or person# or people)) or su (older n1 (adult# or m?n or wom?n or person# or people)) |
| s9 | tx (age# n3 (over or older) n2 (50 or 55 or 60 or 65 or 70 or 75 or 80 or 85 or 90 or 95)) |
| s8 | tx sexagenarian or septuagenarian or octogenarian or nonagenarian or centenarian |
| s7 | ti (gerontolog*) or ab (gerontolog*) or su (gerontolog*) |
| s6 | (mh "geriatrics") |
| s5 | (mh "aged+") |
| s4 | (s1 and s2) or s3 |
| s3 | tx ((protein* n3 (breakdown or casein or intake or optimal or quality or recommendations or requirements or soya or supplement* or synthesis or turnover or whey)) or "net protein balance" or "energy balance" or "metabolic adaptation") |
| s2 | (mh "dietary supplements") |
| s1 | (mh "dietary proteins+") |
| CINAHL literature search. Interface: EBSCO | |
| s21 | s17 not s20 |
| s20 | s18 or s19 |
| s19 | (mh "animals") |
| s18 | pt commentary or letter or editorial |
| s17 | s4 and s12 and s16 |
| s16 | s13 or s14 or s15 |
| s15 | tx meta analys* or metaanaly* or (systematic n1 (review or overview)) |
| s14 | (mh "meta analysis") |
| s13 | (mh "literature review+") or (mh “systematic review”) |
| s12 | s5 or s6 or s7 or s8 or s9 or s10 or s11 |
| s11 | ti (elderly or geriatric* or senior) or ab (elderly or geriatric* or senior) or su (elderly or geriatric* or senior) |
| s10 | ti (older n1 (adult# or m?n or wom?n or person# or people)) or ab (older n1 (adult# or m?n or wom?n or person# or people)) or su (older n1 (adult# or m?n or wom?n or person# or people)) |
| s9 | tx (age# n3 (over or older) n2 (50 or 55 or 60 or 65 or 70 or 75 or 80 or 85 or 90 or 95)) |
| s8 | tx sexagenarian or septuagenarian or octogenarian or nonagenarian or centenarian |
| s7 | ti (gerontolog*) or ab (gerontolog*) or su (gerontolog*) |
| s6 | (mh "geriatrics") |
| s5 | (mh "aged+") |
| s4 | (s1 and s2) or s3 |
| s3 | tx ((protein* n3 (breakdown or casein or intake or optimal or quality or recommendations or requirements or soya or supplement* or synthesis or turnover or whey)) or "net protein balance" or "energy balance" or "metabolic adaptation") |
| s2 | (mh "dietary supplements") |
| s1 | (mh "dietary proteins+") |

[**Appendix 3.** Showing critical appraisal of included systematic reviews and meta-analyses using the AMSTAR 2 tool](#_Table_6._AMSTAR)

| AMASTAR 2 question | | | | | | | | | | | | | | | | | |
| --- | --- | --- | --- | --- | --- | --- | --- | --- | --- | --- | --- | --- | --- | --- | --- | --- | --- |
| Author/Data | 1 | 2 | 3 | 4 | 5 | 6 | 7 | 8 | 9 | 10 | 11 | 12 | 13 | 14 | 15 | 16 | Overall quality review |
| Avenell et al. (1) | YES | YES | NO | YES | YES | YES | YES | YES | YES | YES | YES | YES | YES | YES | YES | YES | High quality |
| Beaudart et al. (2) | YES | YES | NO | YES | YES | NO | NO | YES | YES | YES | YES | YES | YES | YES | YES | YES | Low quality |
| Liao et al. (3) | YES | YES | NO | YES | YES | YES | NO | YES | YES | NO | YES | NO | YES | YES | YES | YES | Low quality |
| Oktaviana et al. (4) | YES | YES | NO | YES | NO | YES | NO | YES | YES | NO | YES | YES | YES | YES | YES | YES | Low quality |
| Tu et al. (5) | YES | YES | NO | YES | YES | NO | NO | YES | YES | NO | YES | NO | YES | YES | YES | YES | Low quality |
| Cheng et al. (6) | YES | NO | NO | YES | NO | NO | NO | YES | YES | NO | YES | YES | YES | YES | YES | YES | Critically low quality |
| Chang (7) | YES | YES | NO | NO | YES | NO | NO | YES | YES | NO | YES | YES | YES | YES | YES | YES | Critically low quality |
| Cuyul-Vasquez et al. (8) | YES | PY | NO | NO | YES | YES | YES | PY | YES | NO | YES | NO | YES | YES | NO | YES | Critically low quality |
| Finger et al. (9) | YES | NO | NO | YES | YES | YES | NO | YES | YES | NO | YES | NO | NO | YES | NO | YES | Critically low quality |
| Liao et al. (10) | YES | YES | NO | YES | YES | YES | NO | YES | YES | YES | YES | YES | NO | NO | YES | YES | Critically low quality |
| Hettiarachchi et al. (11) | YES | PY | NO | NO | YES | YES | NO | YES | YES | NO | YES | YES | YES | YES | YES | YES | Critically low quality |
| Hidayat et al. (12) | YES | NO | NO | YES | YES | YES | NO | YES | YES | NO | YES | NO | NO | YES | YES | NO | Critically low quality |
| Hou et al. (13) | YES | NO | NO | NO | YES | YES | NO | YES | YES | NO | YES | NO | NO | YES | NO | YES | Critically low quality |
| Kaminska et al. (14) | YES | NO | NO | NO | YES | YES | NO | YES | YES | YES | YES | YES | YES | YES | YES | YES | Critically low quality |
| Dewansingh et al. (15) | YES | NO | NO | NO | YES | YES | NO | YES | YES | NO | YES | NO | YES | YES | YES | YES | Critically low quality |
| Eglseer et al. (16) | YES | YES | NO | YES | YES | YES | NO | YES | YES | NO | YES | NO | NO | NO | NO | YES | Critically low quality |
| Gomes-Neto et al. (17) | YES | NO | NO | YES | YES | YES | NO | YES | YES | NO | YES | NO | NO | YES | NO | YES | Critically low quality |
| Kirwan et al. (18) | YES | YES | NO | NO | YES | YES | NO | YES | YES | NO | YES | NO | NO | YES | YES | YES | Critically low quality |
| Kwon et al. (19) | NO | NO | NO | PY | NO | NO | NO | NO | YES | NO | YES | YES | YES | YES | YES | YES | Critically low quality |
| Labata-Lezaun et al. (20) | YES | YES | NO | YES | YES | NO | NO | YES | YES | NO | YES | NO | NO | YES | YES | YES | Critically low quality |
| Li ML et al. (21) | YES | YES | NO | NO | YES | YES | NO | YES | YES | NO | YES | YES | YES | YES | YES | YES | Critically low quality |
| Li L et al. (22) | YES | YES | NO | YES | NO | NO | NO | YES | YES | NO | YES | NO | NO | NO | NO | YES | Critically low quality |
| Liao et al. (23) | YES | YES | NO | YES | NO | NO | NO | NO | YES | NO | YES | NO | NO | YES | YES | YES | Critically low quality |
| Luo et al. (24) | YES | NO | NO | YES | YES | YES | NO | YES | YES | NO | YES | NO | NO | YES | YES | NO | Critically low quality |
| Martin-Cantero et al. (25) | YES | YES | NO | NO | YES | YES | NO | YES | YES | NO | YES | NO | NO | YES | NO | YES | Critically low quality |
| Nasimi et al. (26) | YES | YES | NO | NO | YES | YES | NO | YES | YES | NO | YES | YES | YES | YES | YES | YES | Critically low quality |
| Ren et al. (27) | YES | NO | NO | NO | YES | YES | NO | YES | YES | NO | YES | NO | NO | NO | YES | YES | Critically low quality |
| Xu et al. (28) | YES | NO | NO | YES | NO | YES | NO | YES | YES | NO | YES | YES | YES | YES | YES | YES | Critically low quality |
| Stoodley et al. (29) | YES | YES | NO | NO | YES | YES | NO | YES | YES | NO | YES | YES | YES | YES | YES | YES | Critically low quality |
| ten Haaf et al. (30) | YES | NO | NO | YES | YES | YES | NO | YES | YES | NO | YES | NO | YES | YES | YES | YES | Critically low quality |
| Tieland et al. (31) | YES | NO | NO | YES | NO | YES | NO | YES | YES | NO | YES | NO | NO | YES | YES | YES | Critically low quality |
| Veronese et al. (32) | YES | NO | NO | NO | YES | YES | NO | NO | YES | NO | YES | NO | NO | YES | YES | YES | Critically low quality |
| Whaikid et al. (33) | YES | YES | NO | NO | NO | YES | NO | YES | YES | NO | YES | NO | YES | YES | YES | YES | Critically low quality |

Legend: PY = Partially yes

**Appendix 4.** AMSTAR-2 Critical Checklist

Reviews were rated according to the number of weaknesses (“partially yes” or “no” ratings) in “critical” and “non-critical” checklist items

Critical items were items:

2 – “If the report of the review contained an explicit statement that the review methods were established prior to the conduct of the review and justified any significant deviations from the protocol”;

4 – “If the review authors used a comprehensive literature search strategy”;

7 – “If the review authors provided a list of excluded studies and justified the exclusions”;

9 – “If the review authors used a satisfactory technique for assessing the risk of bias (RoB) in individual studies that were included in the review”;

11 – “If meta-analysis was performed did the review authors use appropriate methods for statistical combination of results?”

13 – “Whether the review authors accounted for RoB in individual studies when interpreting/discussing the results of the review” and

15 – “If they performed quantitative synthesis did the review authors carry out an adequate investigation of publication bias (small study bias) and discuss its likely impact on the results of the review?”.

Reviews were categorised as:

- high quality if they had no critical weaknesses and no or one non-critical weakness;
- moderate quality if they had no critical weaknesses and more than one non-critical weakness;
- low quality if one critical weakness with or without non-critical weakness; and
- critically-low quality if more than one critical weakness with or without non-critical weaknesses.

**Appendix 5.** Characteristics of the reviews included

| First Author | Address of the first author | AMSTAR-2 quality | Date searched | Participant analysed | Intervention/duration | Comparator | Number of studies/ participants | Outcome |
| --- | --- | --- | --- | --- | --- | --- | --- | --- |
| Avenell 2016 (1) | UK | High | 11/2015 | Older people in hospital without exercise (Patients with hip fracture). | Protein dose: 13 – 20 g/d  Duration: 3 – 12 mo | No protein | 4/ (361) | - Mortality |
|  |  |  |  |  |  |  | 2/ (223) | - Unfavourable outcome (death/complication) |
| Beaudart 2018 (2) | Belgium | Low | 02/2016 | Community-dwelling healthy older people, older postmenopausal women, and people with pre-frailty or frailty. | Protein dose: 20 – 45 g/d  Duration: 24 wk – 2 yr | Placebo | 4/ (179) | - Lean body mass |
|  |  |  |  |  |  |  | 4/ (243) | - Appendicular lean mass |
|  |  |  |  |  |  |  | 3/ (410) | - Handgrip strength |
|  |  |  |  |  |  |  | 3/ (231) | - Knee extension strength |
| Chang 2023 (7) | Republic of Korea | Critically low | 12/2022 | Older people with sarcopenia. | Whey protein dose: 20 – 22 g/d  Duration: 8 – 13 wk | Isocaloric supplementation | 3/ (380) | - Muscle mass |
|  |  |  |  |  |  |  | 3/ (637) | - Handgrip strength |
|  |  |  |  |  |  |  | 3/ (380) | - Short physical performance battery |
|  |  |  |  |  | Whey protein dose: 20 – 22 g/plus RET  Duration: 8 – 13 wk | RET / Isocaloric supplementation | 3/ (127) | - Muscle mass |
|  |  |  |  |  |  |  | 3/ (380) | - Handgrip strength |
|  |  |  |  |  |  |  | 3/ (127) | - Short physical performance battery |
| Cheng 2018 (6) | Australia | Critically low | 06/2016 | Older people with malnutrition, frailty, sarcopenia, dependency, or acute or chronic conditions (including hospitalised patients) with or without rehabilitation exercise. | Protein dose: 8·5 –27·6 g/ Twice-three/d  Duration: 2 – 24 mo | Supplements of lower protein, placebo, or usual care. | 16/ (970) | - Fat-free mass |
|  |  |  |  |  |  |  | 29/ (1940) | - Muscle strength |
|  |  |  |  |  |  |  | 34/ (3396) | - Physical function |
| Cuyul-Vasquez (8) | Chile | Critically low | 01/2023 | Community-dwelling older people with sarcopenia. | Whey protein: 10 – 35 g/d  Frequency: 1 – 3 times/d  Duration: 8 – 24 wk | RET with or without a placebo  Frequency: 2 – 5 times/wk  Duration of session: 20 – 60 min | 5/ (452) | - Appendicular skeletal muscle mass |
|  |  |  |  |  |  |  | 5/ (452) | - Appendicular skeletal muscle mass index |
|  |  |  |  |  |  |  | 5/ (452) | - Muscle strength |
| Dewansingh (15) | Netherlands | Critically low | 03/2016 | Community-dwelling healthy older people, and those with pre-frailty or frailty, mobility limitations or type 2 diabetes mellitus. Hospitalised patients. | Dairy, or dairy-specific components (not mandatory to be dairy based). Dose: 20 g 3 times/wk – 40 g/d  Plus: RET  Duration: 10 d – 6 mo | RET / Placebo | 8/ (474) | - Lean body mass |
|  |  |  |  |  |  |  | 6/ (417) | - Leg strength |
| Eglseer (16) | Austria | Critically low | 09/2021 | Community-dwelling older people with sarcopenia and obesity. | Protein dose: 12 – 14 g/3 times/wk  Any nutritional and/or exercise intervention with a duration of at least 8 wk | RET/Aerobic exercise 3 times/wk for 1h/session | 3/ (148) | - Lean body mass |
| Finger (9) | Brazil | Critically low | 01/2014 | Older people with sarcopenia, frailty or pre-frail, or mobility limitations. | Protein dose: 6 – 40 g/d  Supplementation or modified diet with increased protein content.  Plus, RET 3 sessions/ wk  Duration: 12 – 72 wk | RET alone (without supplementation) or to exercise combined with non-protein placebo supplementation | 9/ (462) | - Muscle mass |
|  |  |  |  |  |  |  | 8/ (462) | - Lean body mass |
|  |  |  |  |  |  |  | 8/ (462) | - Muscle strength |
| Gomes-Neto (17) | Brazil | Critically low | 06/2015 | Health community-dwelling | Whey protein dose: 20 – 40 g/d  Plus, RET 3 sessions/wk  Duration: 12 – 24 wk | RET / Placebo | 3/ (249) | - Knee extension strength |
|  |  |  |  |  |  |  | 2/ (98) | - Leg press strength |
| Hettiarachchi (11) | Australia | Critically low | 03/2023 | Older people in a mixed setting (community-dwelling/institutionalised).  Healthy and chronic illnesses. | Protein dose: 13 – 44 g  Frequency: variable (1-3 times/d)  Duration: 12 – 24 wk | No supplementation or have a placebo product, without additional protein. | 30/ (3360) | - Muscle mass |
| Hidayat (12) | China | Critically low | 09/2016 | Community-dwelling healthy older people and people with frailty, sarcopenia, sarcopenic obesity, or limited mobility. | Protein dose: 10 trials = 4.2 – 40 g/d, 2 trials = 0.3 - 0.8 g/kg/d  Plus, RET  Duration:12 – 24 wk | RET/Placebo (isocaloric products, flavoured beverages, low protein diet, and carbohydrate beverages) | 10/ (574) | - Lean body mass |
| Hou (13) | China | Critically low | 05/2018 | Community-dwelling healthy older people and those with sarcopenia, obesity, frailty, or limited mobility. | Protein dose: 13.2 g – 40 g 3 times/wk  Plus, RET 2 – 4 times per wk  Duration: 10 – 72 wk | RET 1-4 times per wk / placebo | 6/ not stated | - Handgrip strength |
|  |  |  |  |  |  |  | 11/ not stated | - Knee extension strength |
|  |  |  |  |  |  |  | 5/ not stated | - Leg press strength |
|  |  |  |  |  |  |  | 9/ not stated | - Gait speed |
|  |  |  |  |  |  |  | 4/ not stated | - Timed up and go test |
|  |  |  |  |  |  |  | 7/ not stated | - Chair rise test |
|  |  |  |  |  |  |  | 13/ not stated | - Lean body mass |
|  |  |  |  |  |  |  | 10/ not stated | - Appendicular skeletal mass |
| Kaminska (14) | Poland | Critically low | 12/2022 | Older people with sarcopenia | Protein dose: 20 – 80 g/d  Duration: 4 – 26 wk | Placebo | 4/ not stated | - Appendicular skeletal muscle mass |
|  |  |  |  |  |  |  | 7/ not stated | - Handgrip strength |
|  |  |  |  |  |  |  | 4/ not stated | - Short physical performance battery |
|  |  |  |  |  |  |  | 7/ not stated | - Chair and stand test |
| Kirwan (18) | UK | Critically low | 07/2021 | Community-dwelling healthy older people and those with frailty or sarcopenia. | Protein dose: 6 – 63 g/d  Duration: 10 – 104 wk | Low protein | 7/ (223) | - Total lean body mass |
|  |  |  |  |  |  |  | 5/ (150) | - Appendicular lean mass |
|  |  |  |  |  |  |  | 7/ (223) | - Handgrip strength |
|  |  |  |  |  | Protein dose: 6 – 63 g/d,  Plus, RET  Duration: 10 – 104 wk | RET /low protein | 5/ (85) | - Appendicular lean mass |
|  |  |  |  |  |  |  | 14/ (266) | - Lean body mass |
|  |  |  |  |  |  |  | 4/ (97) | - Handgrip strength |
|  |  |  |  |  |  |  | 7/ (132) | - Knee extension strength |
| Kwon (19) | South Korea | Critically low | 05/2023 | Community-dwelling older people with sarcopenia. | Protein dose: 10 – 40 g/d  Plus, physical exercises.  One RCT used leucine (1 g), arginine (1.5 g), and vitamin D 300 IU complex without whey protein  Only one RCT did not include exercise in their intervention.  Duration: 4 wk – 12 mo | RET / Multicomponent exercise / placebo.  Frequency: variable | 5/ (541) | - Appendicular skeletal muscle mass |
|  |  |  |  |  |  |  | 6/ (715) | - Handgrip strength |
|  |  |  |  |  |  |  | 5/ (667) | - Short physical performance battery |
| Labata-Lezaun (20) | Spain | Critically low | 06/2020 | Healthy older people. | Whey protein supplementation dose: 15 – 35 g/d  Plus, RET 2 – 3 sessions/wk  Duration: 10 – 24 wk | RET plus a placebo or no placebo | 5/ (137) | - Upper limb strength |
|  |  |  |  |  |  |  | 14/ (589) | - Lower limb strength |
|  |  |  |  |  |  |  | 4/ (182) | - Handgrip strength |
|  |  |  |  |  |  |  | 5/ (182) | - Gait speed |
|  |  |  |  |  |  |  | 3/ (119) | - Short physical performance battery |
|  |  |  |  |  |  |  | 7/ (453) | - Chair rise test |
| Li L (22) | China | Critically low | 07/2021 | Older people with sarcopenia. | Protein supplementation dose: 3 g – 40 g/d  Plus, exercise 2 – 3 sessions/wk  Duration: 12 – 48 wk | Exercise alone or with a placebo | 14/ (888) | - Muscle mass |
|  |  |  |  |  |  |  | 10/ (676) | - Upper extremity strength |
|  |  |  |  |  |  |  | 6/ (359) | - Lower extremity strength |
|  |  |  |  |  |  |  | 9/ (538) | - Gait speed |
| Li ML (21) | China | Critically low | 06/2023 | Community-dwelling and hospitalised older people diagnosed with sarcopenia. | Protein dose: 9.6 – 40 g/intake.  Frequency: variable  Duration: 8 wk – 18 mo | Isocaloric placebo / routine consultation | 4/ (530) | - Appendicular skeletal muscle mass |
|  |  |  |  |  |  |  | 3/ (274) | - Appendicular skeletal muscle mass index |
|  |  |  |  |  |  |  | 2/ (119) | - Handgrip strength |
|  |  |  |  |  |  |  | 2/ (187) | - Gait speed |
|  |  |  |  |  |  |  | 3/ (451) | - 5 Chair stand test |
|  |  |  |  |  |  |  | 6/ (918) | - Short physical performance battery |
|  |  |  |  |  | Protein dose: 9.6 – 40 g/intake.  Plus, RET  Frequency: variable  Duration: 8 wk – 18 mo | RET / placebo | 2/ (239) | - Appendicular skeletal muscle mass index |
|  |  |  |  |  |  |  | 4/ (272) | - Muscle mass |
|  |  |  |  |  |  |  | 2/ (119) | - Handgrip strength |
| Liao 2017 (23) | Taiwan | Critically low | 05/2016 | Older people with obesity | Protein dose: 10 – 35 g/d  Plus, RET  Duration: 4 – 16 wk | RET plus a placebo | 16/ 802 | - Lean body mass |
|  |  |  |  |  |  |  | 13/ (668) | - Leg Strength |
|  |  |  |  |  |  |  | 6/ (171) | - Upper body strength |
|  |  |  |  |  |  |  | 6/ (357) | - Handgrip strength |
|  |  |  |  |  |  |  | 8/ (553) | - Gait speed |
|  |  |  |  |  |  |  | 2/ (103) | - Short physical performance battery |
|  |  |  |  |  |  |  | 3/ (215) | - Timed up and go test |
|  |  |  |  |  |  |  | 7/ (453) | - Chair rise test |
| Liao 2020 (3) | Taiwan | Low | Not Recorded | Community-dwelling people following hip or knee arthroplasty, or hip osteoarthritis. | Protein dose: 8 – 40 g/d  Plus, RET alone or a multi-component exercise regime (MET) that comprises RET, aerobic exercise, balance training, and physical activity training  Duration: 2 – 4 wk | Placebo supplement, PS alone, ET alone, or none of the above (i.e., regular care) | 4/ (114) | - Muscle mass |
|  |  |  |  |  |  |  | 3/ (98) | - Muscle strength |
|  |  |  |  |  |  |  | 2/ 67 | - Walking capability |
|  |  |  |  |  |  |  | 2/ 67 | - Timed up and go test |
| Liao 2024 (10) | Taiwan | Critically low | 07/2023 | Community-dwelling / Hospitalised and Institutionalised older people with acute and chronic illnesses. | Different protein types and doses: 10 – 80 g/d  Plus, RET  Duration: ≤12 wk – 12 mo | RET with or without a placebo | Not stated/ not stated | - Muscle mass |
|  |  |  |  |  |  |  | 37/ not stated | - Handgrip strength |
|  |  |  |  |  |  |  | 56/ not stated | - Leg strength |
|  |  |  |  |  |  |  | 35/ not stated | - Walking speed |
|  |  |  |  |  |  |  | 31/ not stated | - Chair rise test |
|  |  |  |  |  |  |  | 13/ not stated | - Short physical performance test |
| Luo (24) | China | Critically low | 07/2016 | Older people with sarcopenia, in community. | Proteins dose: 6 – 40 g/d  plus, mixed exercise Duration: 12 – 16 wk | Only received exercise intervention  (mixed exercise) | 4/ (272) | - Muscle mass |
|  |  |  |  |  |  |  | 3/ (207) | - Usual walk speed |
| Martin-Cantero (25) | Australia | Critically low | 11/2017 | Community-dwelling healthy older people or with sarcopenia, pre-frailty, or frailty.  Hospitalised patients with malnutrition, hip fractures, acute stroke, and risk of malnutrition. | Protein dose: 25 – 30 g/meal.  Duration: 1 – 104 wk | Placebo product | 9/ (742) | - Mixture of Measures |
| Nasimi 2023 (26) | Iran | Critically low | 06/2022 | Healthy older people.  Older people with sarcopenia or frailty and without any other overt disease that may affect the outcome. | Whey protein dose: 15 – 40 g/d  Duration: 12 wk – 2 yr | No intervention or isocaloric and non-isocaloric placebo | 16/ not stated | - Lean body mass |
|  |  |  |  |  |  |  | 15/ not stated | - Appendicular lean mass |
|  |  |  |  |  |  |  | 32/ not stated | - Muscle strength |
|  |  |  |  |  |  |  | 7/ not stated | - Short physical performance battery |
|  |  |  |  |  |  |  | 9/ not stated | - Gait speed |
|  |  |  |  |  | Whey protein dose: 15 – 40 g/d  P[us RET  Duration: 12 wk – 2 yr | RET | Not stated / not stated | - Lean body mass |
|  |  |  |  |  |  |  | Not stated / not stated | - Muscle strength |
|  |  |  |  |  |  |  | Not stated / not stated | -Physical function |
| Oktaviana (4) | Australia | Low | 07/2019 | Older people with frailty. | Protein dose: 12 g/ wk – 32 g/d  Duration: 8 – 24 wk | Usual care | 3/ (161) | - Lean body mass |
|  |  |  |  |  |  |  | 3/ (161) | - Handgrip strength |
|  |  |  |  |  |  |  | 3/ (161) | - Lower extremity strength |
|  |  |  |  |  |  |  | 3/ (161) | - Leg press strength |
|  |  |  |  |  |  |  | 3/ (161) | - Short physical performance battery |
|  |  |  |  |  |  |  | 3/ (161) | - Gait velocity |
| Ren (27) | China | Critically low | 12/2023 | Healthy older people.  Older people with sarcopenia or frailty. | Protein dose: 1.2 g/d – 32.4 g twice/d  Duration: 12 – 48 wk | Placebo | Not stated/ (254) | - Appendicular skeletal muscle mass |
|  |  |  |  |  |  |  | Not stated/ (1242) | - Handgrip strength |
|  |  |  |  |  |  |  | Not stated/ (1245) | - Gait speed |
|  |  |  |  |  |  |  | Not stated/ (620) | - Timed up and go test |
|  |  |  |  |  |  |  | 17/ (1494) | - Short physical performance test |
| Stoodley (29) | Australia | Critically low | 01/2023 | Community-dwelling older people. | Plant protein: 0.6 – 60 g/d  Duration: 12 wk – 1yr | Placebo | 9/ (405) | - Lean mass |
|  |  |  |  |  |  |  | 4/ (125) | - Knee extension |
|  |  |  |  |  |  |  | 3/ (200) | - Gait speed |
|  |  |  |  |  | Plant protein: 0.6 – 60 g/d  Plus, exercise  Duration: 12 wk – 1yr | Placebo / exercise | 6/ (270) | - Lean body mass |
| ten Haaf (30) | Netherlands | Critically low | 05/2018 | Non-frail community-dwelling older people. | Protein dose:13.2 – 125 g/d  Duration: 6 – 104 wk | Placebo | 15/ not stated | - Lean body mass |
|  |  |  |  |  | Protein dose:13.2 – 125 g/d  Plus, RET  Duration: 6 – 104 wk | RET | 15/ not stated | - Muscle cross-sectional area (thigh) |
|  |  |  |  |  |  |  | 7/ not stated | - Upper body strength |
|  |  |  |  |  |  |  | 7/ not stated | - Lower extremity muscle strength |
|  |  |  |  |  |  |  | Not stated/ not stated | - Gait speed |
|  |  |  |  |  |  |  | Not stated/ not stated | - Chair rise test |
| Tieland (31) | Netherlands | Critically low | 07/2016 | Community-dwelling healthy older people, and those with frailty, sarcopenia, or  type 2 diabetes mellitus. | Protein dose: 6 – 30 g/d  Duration: 84 – 730 d | Health education, isocaloric and non-isocaloric placebo capsules or non-isocaloric carbohydrate containing drinks | 8/ (412) | - Lean body mass |
|  |  |  |  |  |  |  | 8/ (121) | - Leg press strength |
|  |  |  |  |  |  |  | 8/ (121) | - Leg extension strength |
|  |  |  |  |  |  |  | 8/ (318) | - Handgrip strength |
| Tu (5) | Taiwan | Low | 05/2021 | Community-dwelling healthy people and community-dwelling or hospitalised people with frailty, sarcopenia or sarcopenic obesity, or type 2 diabetes mellitus. | Protein dose: 3 – 40 g. One-two times/d  Duration: 8 wk – 12 mo | Placebo | 6/ (470) | - Appendicular skeletal muscle mass index |
|  |  |  |  |  |  |  | 10/ (764) | - Handgrip strength |
|  |  |  |  |  |  |  | 4/ (276) | - Gait speed |
|  |  |  |  |  |  |  | 5/ (403) | - Chair rise test |
|  |  |  |  |  |  |  | 7/ (543) | - Short physical performance battery |
| Veronese (32) | Italy | Critically low | 09/2018 | Community-dwelling healthy older people. People with frailty, sarcopenia, vulnerability, and previous falls. No hospital studies. | Protein dose: 15 g. Twice/d  Duration: 7 – 108 wk | Placebo | 3/ (286) | - Timed up and go test |
|  |  |  |  |  |  |  | 3/ (182) | - Gait speed |
|  |  |  |  |  |  |  | 2/ (120) | - Chair rise test |
|  |  |  |  |  |  |  | 4/ (344) | - Leg extension strength |
|  |  |  |  |  |  |  | 2/ (224) | - Leg flexion |
|  |  |  |  |  |  |  | 3/ (159) | - Leg press strength |
|  |  |  |  |  |  |  | 2/ (101) | - Maximal isometric strength |
|  |  |  |  |  |  |  | 7/ (535) | - Handgrip strength |
| Whaikid 2024 (33) | Thailand | Critically low | 01/2023 | Community-dwelling older people with sarcopenia | Protein dose: 8.61 – 40 g/d  Plus, RET  Duration: 10 – 24 wk | Usual care | 2/ (232) | - Muscle mass |
|  |  |  |  |  |  |  | 4/ (272) | - Handgrip strength |
|  |  |  |  |  |  |  | 6/ (359) | - 5 Chair stand test |
|  |  |  |  |  |  |  | 2/ (91) | - Gait speed |
| Xu 2014 (28) | China | Critically low | 06/2014 | Community-dwelling healthy older people, and older people with pre-frailty or frailty, or type 2 diabetes mellitus. | Protein dose: 15 – 45 g. Once-twice/d  Duration: 10 d – 6 mo | Placebo | 6/ (394) | - Lean body mass |
|  |  |  |  |  |  |  | 3/ (207) | - Leg press strength |
|  |  |  |  |  |  |  | 4/ (274) | - Leg extension strength |

Abbreviations: d=day; mo=month; g=grams; RET= resistance exercise training; wk=week; yr=year

**Appendix 6.** Results of the GRADE Certainty Assessment

| **Review (First Author)** | **Review design** | **Risk of bias** | **Inconsistency** | **Indirectness** | **Imprecision** | **Other considerations** | **Outcome measured** | **Certainty** |
| --- | --- | --- | --- | --- | --- | --- | --- | --- |
|  |  |  | **Measures of mass** | | | |  |  |
| **Healthy community-dwelling older people, results from studies without concomitant exercise** | | | | | | | | |
| ten Haaf | SR/MA | Serious^a^ | Very serious^d^ | Not serious | Not serious | None | Lean body mass | ⨁◯◯◯  Very low |
| Stoodley | SR/MA | Serious^a^ | Very serious^d^ | Not serious | Very serious^c^ | None | Lean body mass | ⨁◯◯◯  Very low |
| Kirwan | SR/MA | Serious^a^ | Not serious | Not serious | Not serious | None | Appendicular lean mass | ⨁⨁⨁◯  Moderate |
| **Healthy community-dwelling older people, results from studies with concomitant exercise** | | | | | | | | |
| ten Haaf | SR/MA | Serious^a^ | Very serious^d^ | Not serious | Not serious | None | Lean body mass | ⨁◯◯◯  Very low |
| Stoodley | SR/MA | Serious^a^ | Not serious | Not serious | Very serious^c^ | None | Lean body mass | ⨁◯◯◯  Very low |
| Hidayat | SR/MA | Serious^a^ | Not serious | Not serious | Serious^c^ | None | Lean body mass | ⨁◯◯◯  Very low |
| **Healthy community-dwelling older people, results from studies with and without concomitant exercise** | | | | | | | | |
| Stoodley | SR/MA | Serious^a^ | Not serious | Not serious | Very serious^c^ | None | Lean body mass | ⨁◯◯◯  Very low |
| Nasimi | SR/MA | Serious^a^ | Not serious | Not serious | Serious^c^ | None | Lean body mass | ⨁◯◯◯  Very low |
| Kirwan | SR/MA | Serious^a^ | Not serious | Not serious | Not serious | None | Appendicular lean mass | ⨁⨁⨁◯  Moderate |
| **Community-dwelling adults with long-term conditions, results from studies without concomitant exercise** | | | | | | | | |
| Hettiarachchi | SR/MA | Serious^a^ | Not serious | Not serious | Not serious | None | Muscle mass | ⨁⨁⨁◯  Moderate |
| Li ML | SR/MA | Serious^a^ | Not serious | Not serious | Not serious | None | Appendicular skeletal muscle mass index | ⨁⨁⨁◯  Moderate |
| Kirwan | SR/MA | Serious^a^ | Not serious | Not serious | Not serious | None | Appendicular lean mass | ⨁⨁⨁◯  Moderate |
| **Community-dwelling adults with long-term conditions, results from studies with concomitant exercise** | | | | | | | | |
| Eglseer | SR/MA | Serious^a^ | Not serious | Not serious | Serious^c^ | None | Lean body mass | ⨁◯◯◯  Very low |
| Liao 2020 | SR/MA | Serious^a^ | Not serious | Serious | Serious^c^ | None | Pooled UL & LL measures of mass & volume | ⨁◯◯◯  Very low |
| Cuyul-Vasquez | SR/MA | Serious^a^ | Not serious | Not serious | Not serious | None | Appendicular skeletal muscle mass index | ⨁⨁⨁◯  Moderate |
| Li ML | SR/MA | Serious^a^ | Not serious | Not serious | Not serious | None | Appendicular skeletal muscle mass index | ⨁⨁⨁◯  Moderate |
| Li L | SR/MA | Serious^a^ | Not serious | Not serious | Not serious | None | Mixture of measures | ⨁⨁⨁◯  Moderate |
| Kirwan | SR/MA | Serious^a^ | Not serious | Not serious | Not serious | None | Appendicular lean mass | ⨁⨁⨁◯  Moderate |
| Hidayat | SR/MA | Serious^a^ | Not serious | Not serious | Serious^c^ | None | Fat-free mass | ⨁◯◯◯  Very low |
| Luo | SR/MA | Serious^a^ | Not serious | Not serious | Very serious^c^ | None | Muscle mass | ⨁◯◯◯  Very low |
| Hou | SR/MA | Serious^a^ | Not serious | Not serious | Not serious | Publication bias strongly suspected^e^ | Appendicular skeletal muscle mass | ⨁⨁◯◯  Low |
| Whaikid | SR/MA | Serious^a^ | Not serious | Not serious | Not serious | None | Muscle mass | ⨁⨁⨁◯  Moderate |
| **Community-dwelling adults with long-term conditions, results from studies with and without concomitant exercise** | | | | | | | | |
| Chang | SR/MA | Serious^a^ | Not serious | Not serious | Serious^c^ | None | Appendicular skeletal muscle mass | ⨁◯◯◯  Very low |
| Kirwan | SR/MA | Serious^a^ | Not serious | Not serious | Not serious | None | Lean body mass | ⨁⨁⨁◯  Moderate |
| Kaminska | SR/MA | Serious^a^ | Not serious | Not serious | Not serious | None | Appendicular skeletal muscle mass | ⨁⨁⨁◯  Moderate |
| Kwon | SR/MA | Serious^a^ | Not serious | Not serious | Not serious | None | Appendicular skeletal muscle mass | ⨁⨁⨁◯  Moderate |
| Nasimi | SR/MA | Serious^a^ | Not serious | Not serious | Not serious | None | Lean body mass | ⨁⨁⨁◯  Moderate |
| Ren | SR/MA | Serious^a^ | Not serious | Not serious | Not serious | None | Appendicular skeletal muscle mass | ⨁⨁⨁◯  Moderate |
| Oktaviana | SR/MA | Serious^a^ | Not serious | Not serious | Not serious | None | Lean body mass | ⨁⨁⨁◯  Moderate |
| **Older people in hospital, results from studies without concomitant exercise** | | | | | | | | |
| Avenell 2016 | SR/MA | Serious^a^ | Not serious | Not serious | Not serious | None | Mortality | ⨁⨁⨁◯  Moderate |
| Avenell 2016 | SR/MA | Serious^a^ | Not serious | Not serious | Not serious | None | Complications | ⨁⨁⨁◯  Moderate |
| Hettiarachchi | SR/MA | Serious^a^ | Not serious | Not serious | Not serious | None | Muscle mass | ⨁⨁⨁◯  Moderate |
| **Mixed community-dwelling older populations, results from studies without exercise** | | | | | | | | |
| Kirwan | SR/MA | Serious^a^ | Not serious | Not serious | Not serious | None | Appendicular skeletal muscle mass | ⨁⨁⨁◯  Moderate |
| Beaudart | SR/MA | Serious^a^ | Not serious | Not serious | Not serious | None | Appendicular skeletal muscle mass | ⨁⨁⨁◯  Moderate |
| Liao 2024 | NMA | Serious^a^ | Not serious | Not serious | Not serious | Reporting bias detected | Muscle mass-Meat | ⨁⨁◯◯  Low |
| Liao 2024 | NMA | Serious^a^ | Not serious | Not serious | Not serious | Reporting bias detected | Muscle mass-Milk | ⨁⨁◯◯  Low |
| Liao 2024 | NMA | Serious^a^ | Not serious | Not serious | Not serious | Reporting bias detected | Muscle mass-Whey | ⨁⨁◯◯  Low |
| Liao 2024 | NMA | Serious^a^ | Not serious | Not serious | Not serious | Reporting bias detected | Muscle mass-Soy | ⨁⨁◯◯  Low |
| Nasimi | SR/MA | Serious^a^ | Not serious | Not serious | Not serious | None | Lean body mass | ⨁⨁⨁◯  Moderate |
| Hettiarachchi | SR/MA | Serious^a^ | Not serious | Not serious | Not serious | None | Mixture of measures | ⨁⨁⨁◯  Moderate |
| Martin-Cantero | SR/MA | Serious^a^ | Not serious | Not serious | Not serious | None | Mixture of measures | ⨁⨁⨁◯  Moderate |
| Cheng | SR/MA | Serious^a^ | Not serious | Not serious | Not serious | None | Fat-free mass | ⨁⨁⨁◯  Moderate |
| **Mixed community-dwelling older populations, results from studies with exercise** | | | | | | | | |
| Liao 2017 | SR/MA | Serious^a^ | Not serious | Not serious | Not serious | None | Appendicular lean mass | ⨁⨁⨁◯  Moderate |
| Kirwan | SR/MA | Serious^a^ | Not serious | Not serious | Not serious | None | Appendicular skeletal muscle mass | ⨁⨁⨁◯  Moderate |
| Finger | SR/MA | Serious^a^ | Not serious | Not serious | Not serious | None | Muscle mass | ⨁⨁⨁◯  Moderate |
| Hidayat | SR/MA | Serious^a^ | Not serious | Not serious | Serious^c^ | None | Fat-free mass | ⨁◯◯◯  Very low |
| Liao 2024 | NMA | Serious^a^ | Not serious | Not serious | Not serious | Reporting bias detected | Muscle mass-Meat | ⨁⨁◯◯  Low |
| Liao 2024 | NMA | Serious^a^ | Not serious | Not serious | Not serious | Reporting bias detected | Muscle mass-Milk | ⨁⨁◯◯  Low |
| Liao 2024 | NMA | Serious^a^ | Not serious | Not serious | Not serious | Reporting bias detected | Muscle mass-Whey | ⨁⨁◯◯  Low |
| Liao 2024 | NMA | Serious^a^ | Not serious | Not serious | Not serious | Reporting bias detected | Muscle mass-Casein | ⨁⨁◯◯  Low |
| Liao 2024 | NMA | Serious^a^ | Not serious | Not serious | Not serious | Reporting bias detected | Muscle mass-Peanut | ⨁⨁◯◯  Low |
| Liao 2024 | NMA | Serious^a^ | Not serious | Not serious | Not serious | Reporting bias detected | Muscle mass-Soy | ⨁⨁◯◯  Low |
| Nasimi | SR/MA | Serious^a^ | Not serious | Not serious | Not serious | None | Lean body mass | ⨁⨁⨁◯  Moderate |
| **Mixed community-dwelling older populations, results from studies with and without concomitant exercise** | | | | | | | | |
| Nasimi | SR/MA | Serious^a^ | Not serious | Not serious | Not serious | None | Appendicular lean mass | ⨁⨁⨁◯  Moderate |
| Xu | SR/MA | Serious^a^ | Not serious | Not serious | Not serious | None | Lean body mass | ⨁⨁⨁◯  Moderate |
| Kirwan | SR/MA | Serious^a^ | Not serious | Not serious | Not serious | None | Appendicular skeletal muscle mass | ⨁⨁⨁◯  Moderate |
| Dewansingh | SR/MA | Serious^a^ | Not serious | Not serious | Not serious | None | Lean body mass | ⨁⨁⨁◯  Moderate |
| Tu | SR/MA | Serious^a^ | Not serious | Not serious | Not serious | None | Appendicular skeletal muscle mass index | ⨁⨁⨁◯  Moderate |
| Tieland | SR/MA | Serious^a^ | Not serious | Not serious | Not serious | None | Lean body mass | ⨁⨁⨁◯  Moderate |
| Ren | SR/MA | Serious^a^ | Not serious | Not serious | Not serious | None | Appendicular skeletal muscle mass | ⨁⨁⨁◯  Moderate |
|  |  |  | **Measures of strength** | | | |  |  |
| **Healthy community-dwelling older people, results from studies without concomitant exercise** | | | | | | | | |
| ten Haaf | SR/MA | Serious^a^ | Very serious^d^ | Not serious | Not serious | None | Leg extension strength | ⨁◯◯◯ Very low |
| **Healthy community-dwelling older people, results from studies with concomitant exercise** | | | | | | | | |
| Gomes-Neto | SR/MA | Serious^a^ | Very serious^d^ | Not serious | Very serious^c^ | None | Knee extension strength | ⨁◯◯◯  Very low |
| ten Haaf | SR/MA | Serious^a^ | Very serious^d^ | Not serious | Not serious | None | Lower extremity strength | ⨁◯◯◯  Very low |
| **Healthy community-dwelling older people, results from studies with and without concomitant exercise** | | | | | | | | |
| Stoodley | SR/MA | Serious^a^ | Not serious | Not serious | Serious^c^ | None | Knee extension strength | ⨁⨁◯◯  Low |
| Nasimi | SR/MA | Serious^a^ | Not serious | Not serious | Not serious | None | Muscle strength | ⨁⨁⨁◯  Moderate |
| **Community-dwelling adults with long-term conditions, results from studies without concomitant exercise** | | | | | | | | |
| Li ML | SR/MA | Serious^a^ | Not serious | Not serious | Very serious^c^ | None | Handgrip strength | ⨁⨁◯◯  Low |
| Kirwan | SR/MA | Serious^a^ | Not serious | Not serious | Not serious | None | Handgrip strength | ⨁⨁⨁◯  Moderate |
| **Community-dwelling adults with long-term conditions, results from studies with concomitant exercise** | | | | | | | | |
| Chang | SR/MA | Serious^a^ | Not serious | Not serious | Very serious^c^ | None | Handgrip strength | ⨁◯◯◯  Very low |
| Liao 2020 | SR/MA | Serious^a^ | Not serious | Serious | Not serious | None | Operated lower limb | ⨁⨁◯◯  Low |
| Liao 2020 | SR/MA | Serious^a^ | Not serious | Serious | Not serious | None | Un-operated lower limb | ⨁⨁◯◯  Low |
| Cuyul-Vasquez | SR/MA | Serious^a^ | Not serious | Not serious | Not serious | None | Handgrip strength | ⨁⨁⨁◯  Moderate |
| Li ML | SR/MA | Serious^a^ | Not serious | Not serious | Not serious | None | Handgrip strength | ⨁⨁◯◯  Low |
| Whaikid | SR/MA | Serious^a^ | Not serious | Not serious | Not serious | None | Handgrip strength | ⨁⨁⨁◯  Moderate |
| Kirwan | SR/MA | Serious^a^ | Not serious | Not serious | Not serious | None | Handgrip strength | ⨁⨁⨁◯  Moderate |
| Li L | SR/MA | Serious^a^ | Not serious | Not serious | Not serious | None | Lower limb strength | ⨁⨁⨁◯  Moderate |
| Hou | SR/MA | Serious^a^ | Not serious | Not serious | Not serious | Publication bias strongly suspected | Knee extension strength | ⨁⨁◯◯  Low |
| **Community-dwelling adults with long-term conditions, results from studies with and without concomitant exercise** | | | | | | | | |
| Chang | SR/MA | Serious^a^ | Not serious | Not serious | Serious^c^ | None | Handgrip strength | ⨁◯◯◯  Very low |
| Kaminska | SR/MA | Serious^a^ | Not serious | Not serious | Not serious | None | Handgrip strength | ⨁⨁⨁◯  Moderate |
| Kwon | SR/MA | Serious^a^ | Not serious | Not serious | Not serious | None | Handgrip strength | ⨁⨁⨁◯  Moderate |
| Nasimi | SR/MA | Serious^a^ | Not serious | Not serious | Not serious | None | Knee extension and leg press strength | ⨁⨁⨁◯  Moderate |
| Oktaviana | SR/MA | Serious^a^ | Not serious | Not serious | Not serious | None | Leg press strength | ⨁⨁⨁◯  Moderate |
| **Mixed community-dwelling older populations, results from studies without exercise** | | | | | | | | |
| Kirwan | SR/MA | Serious^a^ | Not serious | Not serious | Not serious | None | Handgrip strength | ⨁⨁⨁◯  Moderate |
| Beaudart | SR/MA | Serious^a^ | Not serious | Not serious | Not serious | None | Knee extension strength | ⨁⨁⨁◯  Moderate |
| Liao 2024 | NMA | Serious^a^ | Not serious | Not serious | Not serious | Reporting bias detected | Leg strength-Meat | ⨁⨁◯◯  Low |
| Liao 2024 | NMA | Serious^a^ | Not serious | Not serious | Not serious | Reporting bias detected | Leg strength-Milk | ⨁⨁◯◯  Low |
| Liao 2024 | NMA | Serious^a^ | Not serious | Not serious | Not serious | Reporting bias detected | Leg strength-Soy | ⨁⨁◯◯  Low |
| Liao 2024 | NMA | Serious^a^ | Not serious | Not serious | Not serious | Reporting bias detected | Leg strength-Whey | ⨁⨁◯◯  Low |
| Nasimi | SR/MA | Serious^a^ | Not serious | Not serious | Not serious | None | Muscle strength | ⨁⨁⨁◯  Moderate |
| **Mixed community-dwelling older populations, results from studies with concomitant exercise** | | | | | | | | |
| Labata-Lezaun | SR/MA | Serious^a^ | Very serious^d^ | Not serious | Not serious | None | Lower limb strength | ⨁◯◯◯  Very low |
| Kirwan | SR/MA | Serious^a^ | Not serious | Not serious | Not serious | None | Knee extension strength | ⨁⨁⨁◯  Moderate |
| Nasimi | SR/MA | Serious^a^ | Not serious | Not serious | Not serious | None | Knee extension/Leg press strength | ⨁⨁⨁◯  Moderate |
| Finger | SR/MA | Serious^a^ | Not serious | Not serious | Not serious | None | Muscle strength | ⨁⨁⨁◯  Moderate |
| Liao 2017 | SR/MA | Serious^a^ | Serious^d^ | Not serious | Not serious | None | Lower limb strength | ⨁⨁◯◯  Low |
| Liao 2024 | NMA | Serious^a^ | Not serious | Not serious | Not serious | Reporting bias detected | Leg strength-Casein | ⨁⨁◯◯  Low |
| Liao 2024 | NMA | Serious^a^ | Not serious | Not serious | Not serious | Reporting bias detected | Leg strength-Meat | ⨁⨁◯◯  Low |
| Liao 2024 | NMA | Serious^a^ | Not serious | Not serious | Not serious | Reporting bias detected | Leg strength-Soy | ⨁⨁◯◯  Low |
| Liao 2024 | NMA | Serious^a^ | Not serious | Not serious | Not serious | Reporting bias detected | Leg strength-Milk | ⨁⨁◯◯  Low |
| Liao 2024 | NMA | Serious^a^ | Not serious | Not serious | Not serious | Reporting bias detected | Leg strength-Whey | ⨁⨁◯◯  Low |
| **Mixed community-dwelling older populations, results from studies with and without concomitant exercise** | | | | | | | | |
| Xu | SR/MA | Serious^a^ | Not serious | Not serious | Not serious | None | Leg extension strength | ⨁⨁⨁◯  Moderate |
| Kirwan | SR/MA | Serious^a^ | Not serious | Not serious | Not serious | None | Handgrip strength | ⨁⨁⨁◯  Moderate |
| Kirwan | SR/MA | Serious^a^ | Not serious | Not serious | Not serious | None | Leg extension strength | ⨁⨁⨁◯  Moderate |
| Tu | SR/MA | Serious^a^ | Not serious | Not serious | Not serious | None | Handgrip strength | ⨁⨁⨁◯  Moderate |
| Veronese | SR/MA | Serious^a^ | Not serious | Not serious | Not serious | None | Leg extension strength | ⨁⨁⨁◯  Moderate |
| Ren | SR/MA | Serious^a^ | Not serious | Not serious | Not serious | None | Handgrip strength | ⨁⨁⨁◯  Moderate |
| Nasimi | SR/MA | Serious^a^ | Not serious | Not serious | Not serious | None | Knee extension/Leg press strength | ⨁⨁⨁◯  Moderate |
| Dewansingh | SR/MA | Serious^a^ | Not serious | Not serious | Not serious | None | Leg strength | ⨁⨁⨁◯  Moderate |
| Cheng | SR/MA | Serious^a^ | Not serious | Not serious | Not serious | None | Combined upper and lower limb measures | ⨁⨁⨁◯  Moderate |
| Tieland | SR/MA | Serious^a^ | Not serious | Not serious | Not serious | None | Leg extension strength | ⨁⨁⨁◯  Moderate |
|  |  |  | **Physical performance** | | | |  |  |
| **Healthy community-dwelling older people, results from studies without concomitant exercise** | | | | | | | | |
| ten Haaf | SR/MA | Serious^a^ | Not serious | Not serious | Not serious | None | Chair rise test | ⨁⨁⨁◯  Moderate |
| **Healthy community-dwelling older people, results from studies with concomitant exercise** | | | | | | | | |
| ten Haaf | SR/MA | Serious^a^ | Not serious | Not serious | Not serious | None | Chair rise test | ⨁⨁⨁◯  Moderate |
| Stoodley | SR/MA | Serious^a^ | Not serious | Not serious | Very serious | None | Gait speed | ⨁⨁◯◯  Low |
| **Healthy community-dwelling older people, results from studies with and without concomitant exercise** | | | | | | | | |
| Nasimi | SR/MA | Serious^a^ | Not serious | Not serious | Not serious | None | Composite of all performance outcome | ⨁⨁⨁◯  Moderate |
| **Community-dwelling older people with long-term conditions, results from studies without concomitant exercise** | | | | | | | | |
| Li ML | SR/MA | Serious^a^ | Not serious | Not serious | Serious^c^ | Publication bias strongly suspected^e^ | Short physical performance battery | ⨁◯◯◯  Very low |
| **Community-dwelling older people with long-term conditions, results from studies with concomitant exercise** | | | | | | | | |
| Liao 2020 | SR/MA | Serious^a^ | Not serious | Serious | Serious^c^ | None | Timed up and go test | ⨁◯◯◯  Very low |
| Whaikid | SR/MA | Serious^a^ | Not serious | Not serious | Not serious | None | 5-Chair rise test | ⨁⨁⨁◯  Moderate |
| Li L | SR/MA | Serious^a^ | Not serious | Not serious | Not serious | None | Gait speed | ⨁⨁⨁◯  Moderate |
| Hou | SR/MA | Serious^a^ | Not serious | Not serious | Not serious | Publication bias strongly suspected | Chair rise test | ⨁⨁◯◯  Low |
| **Community-dwelling adults with long-term conditions, results from studies with and without concomitant exercise** | | | | | | | | |
| Chang | SR/MA | Serious^a^ | Not serious | Not serious | Very serious | None | Short physical performance battery | ⨁◯◯◯  Very low |
| Kaminska | SR/MA | Serious^a^ | Not serious | Not serious | Not serious | None | Short physical performance battery | ⨁⨁⨁◯  Moderate |
| Kwon | SR/MA | Serious^a^ | Not serious | Not serious | Serious | None | Short physical performance battery | ⨁◯◯◯  Very low |
| Nasimi | SR/MA | Serious^a^ | Not serious | Not serious | Not serious | None | Composite of all performance outcome | ⨁⨁⨁◯  Moderate |
| Oktaviana | SR/MA | Serious^a^ | Serious^d^ | Not serious | Serious^c^ | None | Short physical performance battery | ⨁◯◯◯  Very low |
| **Mixed community-dwelling older populations, results from studies without exercise** | | | | | | | | |
| Liao 2024 | NMA | Serious^a^ | Not serious | Not serious | Not serious | Reporting bias detected | Short physical performance battery-Whey | ⨁⨁◯◯  Low |
| Nasimi | SR/MA | Serious^a^ | Not serious | Not serious | Not serious | None | Composite of all performance outcome | ⨁⨁⨁◯  Moderate |
| **Mixed community-dwelling older populations, results from studies with concomitant exercise** | | | | | | | | |
| Labata-Lezaun | SR/MA | Serious^a^ | Not serious | Not serious | Not serious | None | Short physical performance battery | ⨁◯◯◯  Very low |
| Liao 2017 | SR/MA | Serious^a^ | Serious^d^ | Not serious | Not serious | None | Short physical performance battery | ⨁⨁◯◯  Low |
| Nasimi | SR/MA | Serious^a^ | Not serious | Not serious | Not serious | None | Short physical performance battery | ⨁⨁⨁◯  Moderate |
| Liao 2024 | NMA | Serious^a^ | Not serious | Not serious | Not serious | Reporting bias detected | Short physical performance battery-Casein | ⨁⨁◯◯  Low |
| Liao 2024 | NMA | Serious^a^ | Not serious | Not serious | Not serious | Reporting bias detected | Short physical performance battery-Milk | ⨁⨁◯◯  Low |
| Liao 2024 | NMA | Serious^a^ | Not serious | Not serious | Not serious | Reporting bias detected | Short physical performance battery-Whey | ⨁⨁◯◯  Low |
| **Mixed community-dwelling older populations, results from studies with and without concomitant exercise** | | | | | | | | |
| Tu | SR/MA | Serious^a^ | Not serious | Not serious | Not serious | Not serious | Short physical performance battery | ⨁⨁⨁◯  Moderate |
| Veronese | SR/MA | Serious^a^ | Not serious | Not serious | Not serious | Not serious | Chair rise test | ⨁⨁⨁◯  Moderate |
| Ren | SR/MA | Serious^a^ | Not serious | Not serious | Not serious | Not serious | Timed up and go test | ⨁⨁⨁◯  Moderate |
| Nasimi | SR/MA | Serious^a^ | Not serious | Not serious | Not serious | Not serious | Short physical performance battery | ⨁⨁⨁◯  Moderate |
| Cheng | SR/MA | Serious^a^ | Not serious | Not serious | Not serious | Not serious | Combined measure | ⨁⨁⨁◯  Moderate |

^a^ Risk of bias detected; ^b^ Unreported heterogeneity; ^c^ Wide confidence intervals; ^d^ Unexplained heterogeneity; ^e^ Language limited to English

**Appendix 7.** Showing excluded reviews with reasons

| **Citation** | **Reason for exclusion** |
| --- | --- |
| 1. Baldwin, C., et al. (2021). "Dietary advice with or without oral nutritional supplements for disease‐related malnutrition in adults." Cochrane Database of Systematic Reviews | No specific results for population aged >60  No specific results for the effect of protein |
| 1. Breen, L., et al. (2007). "Functional benefits of combined resistance training with nutritional interventions in older adults: a review." Geriatrics & Gerontology International 7(4): 326-340 | No specific results for population aged >60 |
| 1. Rabassa-Blanco, J. and I. Palma-Linares (2017). "Effects of protein and branched-chain amino acids supplements in resistance training: A review. [Spanish]." Revista Espanola de Nutricion Humana y Dietetica 21(1): 55-73 | No specific results for population aged >60 |
| 1. Hanach, N. I., et al. (2019). "The impact of dairy protein intake on muscle mass, muscle strength, and physical performance in middle-aged to older adults with or without existing sarcopenia: a systematic review and meta-analysis." Advances in Nutrition 10(1): 59-69 | No specific results for population aged >60 |
| 1. Hudson, J. L., et al. (2018). "Effects of protein supplements consumed with meals, versus between meals, on resistance training-induced body composition changes in adults: a systematic review." Nutrition Reviews 76(6): 461-468 | No specific results for population aged >60 |
| 1. Jespersen, S. E. and J. Agergaard (2021). "Evenness of dietary protein distribution is associated with higher muscle mass but not muscle strength or protein turnover in healthy adults: a systematic review." European Journal of Nutrition 60(6): 3185-3202. | No specific results for population aged >60 |
| 1. Jibril, A. T., et al. (2022). "Effects of Protein and Amino Acid Supplementation on Muscle Mass and Strength in a Healthy Population." Nutrition Today 57(3): 166-178 | No specific results for population aged >60 |
| 1. Langer, G. and A. Fink (2014). "Nutritional interventions for preventing and treating pressure ulcers." Cochrane Database of Systematic Reviews | No specific results for population aged >60 |
| 1. Miller, P. E., et al. (2014). "Effects of Whey Protein and Resistance Exercise on Body Composition: A Meta-Analysis of Randomized Controlled Trials." Journal of the American College of Nutrition 33(2): 163-175 | No specific results for population aged >60 |
| 1. Morton, R. W., et al. (2018). "A systematic review, meta-analysis and meta-regression of the effect of protein supplementation on resistance training-induced gains in muscle mass and strength in healthy adults." British journal of sports medicine 52(6): 376-384. | No specific results for population aged >60 |
| 1. Nunes, E. A., et al. (2022). "Systematic review and meta-analysis of protein intake to support muscle mass and function in healthy adults." Journal of Cachexia, Sarcopenia and Muscle | No specific results for population aged >60 |
| 1. Park, Y. J., et al. (2022). "A review of recent evidence of dietary protein intake and health." Nutrition Research and Practice 16(Suppl 1): S37-S46 | No specific results for population aged >60 |
| 1. Pedersen, A. N., et al. (2013). "Health effects of protein intake in healthy adults: a systematic literature review." Food & nutrition research 57(1): 21245 | No specific results for population aged >60 |
| 1. Potter, J., et al. (1998). "Routine protein energy supplementation in adults: systematic review." BMJ 317(7157): 495-501 | No specific results for population aged >60 |
| 1. Stratton, R. J., et al. (2013). "A systematic review and meta-analysis of the impact of oral nutritional supplements on hospital readmissions." Ageing Research Reviews 12(4): 884-897 | No specific results for population aged >60 |
| 1. Tagawa, R., et al. (2021). "Dose-response relationship between protein intake and muscle mass increase: A systematic review and meta-analysis of randomized controlled trials." Nutrition Reviews 79(1): 66-75 | No specific results for population aged >60 |
| 1. Valenzuela, P. L., et al. (2019). "Does Beef Protein Supplementation Improve Body Composition and Exercise Performance? A Systematic Review and Meta-Analysis of Randomized Controlled Trials." Nutrients 11(6): 25 | No specific results for population aged >60 |
| 1. Wirth, J., et al. (2020). "The role of protein intake and its timing on body composition and muscle function in healthy adults: a systematic review and meta-analysis of randomized controlled trials." The Journal of nutrition 150(6): 1443-1460 | No specific results for population aged >60 |
| 1. Wong, A., et al. (2022). "Effectiveness of dietary counseling with or without nutrition supplementation in hospitalized patients who are malnourished or at risk of malnutrition: A systematic review and meta-analysis." Journal of Parenteral and Enteral Nutrition 46(7): 1502-1521 | No specific results for population aged >60 |
| 1. Van Ruijven I, Abma J, Brunsveld-Reinders A, Stapel S, Van Etten-Jamaludin F, Boirie Y, et al. High protein provision of more than 1.2 g/kg improves muscle mass preservation and mortality in ICU patients: A systematic review and meta-analyses. Intensive Care Medicine Experimental Conference: European Society of Intensive Care Medicine Annual Congress, ESICM. 2023;11(Supplement 1) | No specific results for population aged >60 |

| 1. Beck AM, Dent E, Baldwin C. Nutritional intervention as part of functional rehabilitation in older people with reduced functional ability: a systematic review and meta-analysis of randomised controlled studies. Journal of Human Nutrition & Dietetics. 2016;29(6):733-45. | No specific results for the effect of protein |
| --- | --- |
| 1. Cawood A, Elia M, Stratton R. Systematic review and meta-analysis of the effects of high protein oral nutritional supplements. Ageing research reviews. 2012;11(2):278-96. | Seems to include Vitamin D as protein and poor control group is not isocalorific placebo but rather anything |
| 1. Haines, K. L., et al. (2023). "Optimal Nutrition in the Older Adult: Beneficial Versus Ineffective Supplements." Current Nutrition Reports | No specific results for the effect of protein |
| 1. Komar, B., et al. (2015). "Effects of leucine-rich protein supplements on anthropometric parameter and muscle strength in the elderly: A systematic review and meta-analysis." Journal of Nutrition, Health and Aging 19(4): 437-446 | No specific results for the effect of protein (not solely amino acids) |
| 1. Lee, S. Y., et al. (2022). "Effects of leucine-rich protein supplements in older adults with sarcopenia: A systematic review and meta-analysis of randomized controlled trials." Archives of Gerontology & Geriatrics 102: N.PAG-N.PAG | No specific results for the effect of protein (not solely amino acids) |
| 1. Nowson et al., 2018. The impact of dietary factors on indices of chronic disease in older people: a systematic review | No specific results for the effect of protein |
| 1. Lai WY, Chiu YC, Lu KC, Huang IT, Tsai PS, Huang CJ. Beneficial effects of preoperative oral nutrition supplements on postoperative outcomes in geriatric hip fracture patients A PRISMA-compliant systematic review and meta-analysis of randomized controlled studies. Medicine (United States). 2021;100(47) (no pagination). | Does not answer the question of the specific effect of protein because of the lack of isocalorific control |
| 1. Liao C-D, Lee P-H, Hsiao D-J, Huang S-W, Tsauo J-Y, Chen H-C, et al. Effects of Protein Supplementation Combined with Exercise Intervention on Frailty Indices, Body Composition, and Physical Function in Frail Older Adults. Nutrients. 2018;10(12):1916. | Not a test of the specific effect of protein, nor protein and exercise |
| 1. Liu M, Yang J, Yu X, Huang X, Vaidya S, Huang F, et al. The role of perioperative oral nutritional supplementation in elderly patients after hip surgery. Clinical interventions in aging. 2015:849-58. | No specific results for the effect of protein |
| 1. Palop Montoro, M. V., et al. (2015). "[Sarcopenia intervention with progressive resistance training and protein nutritional supplements]." Nutricion Hospitalaria 31(4): 1481- | No specific results for the effect of protein (not solely amino acids) |
| 1. Malafarina V, Reginster J-Y, Cabrerizo S, Bruyère O, Kanis JA, Martinez JA, et al. Nutritional status and nutritional treatment are related to outcomes and mortality in older adults with hip fracture. Nutrients. 2018;10(5):555. | No specific results for the effect of protein |
| 1. Milne AC, Potter J, Vivanti A, Avenell A. Protein and energy supplementation in elderly people at risk from malnutrition. Cochrane Database of Systematic Reviews. 2009(2). | No specific results for the effect of protein |
| 1. Milne, A. C., et al. (2006). "Meta-analysis: Protein and energy supplementation in older people." Annals of Internal Medicine 144(1): 37-48. | No specific results for the effect of protein |
| 1. Milne, A. C., et al. (2005). "Oral protein and energy supplementation in older people: a systematic review of randomized trials." Nestle Nutrition workshop series Clinical & performance programme. 10: 103-120; discussion 120-125. | No specific results for the effect of protein |
| 1. Milne, A. C., et al. (2003). "Review: Oral protein and energy supplements reduce all-cause mortality in elderly people." Evidence-Based Medicine 8(3): 82 | No specific results for the effect of protein |
| 1. Milne, A. C., et al. (2002). "Protein and energy supplementation in elderly people at risk from malnutrition." Cochrane database of systematic reviews (Online)(3): CD003288 | No specific results for the effect of protein |
| 1. Thomson KH, Rice S, Arisa O, Johnson E, Tanner L, Marshall C, et al. Effectiveness and cost-effectiveness of oral nutritional supplements in frail older people who are malnourished or at risk of malnutrition: a systematic review and meta-analysis. The Lancet Healthy Longevity. 2022;3(10):e654-e66. | No specific results for the effect of protein |
| 1. Trabal J, Farran-Codina A. Effects of dietary enrichment with conventional foods on energy and protein intake in older adults: A systematic review. Nutrition Reviews. 2015;73(9):624-33. | No specific results for the effect of protein |
| 1. Reinders I, Volkert D, de Groot LCPGM, Beck AM, Feldblum I, Jobse I, et al. Effectiveness of nutritional interventions in older adults at risk of malnutrition across different health care settings: Pooled analyses of individual participant data from nine randomized controlled trials. Clinical | No specific results for the effect of protein |
| 1. Rus GE, Porter J, Brunton A, Crocker M, Kotsimbos Z, Percic J, et al. Nutrition interventions implemented in hospital to lower risk of sarcopenia in older adults: A systematic review of randomised controlled trials. Nutrition & Dietetics. 2020;77(1):90-102. | No specific results for the effect of protein |
| 1. van Wijngaarden, J. P., et al. (2020). "Effects of Nutritional Interventions on Nutritional and Functional Outcomes in Geriatric Rehabilitation Patients: A Systematic Review and Meta-Analysis." Journal of the American Medical Directors Association 21(9): 1207-1207. | No specific results for the effect of protein |
| 1. Li W, Wu Z, Liao X, Geng D, Yang J, Dai M, et al. Nutritional management interventions and multi-dimensional outcomes in frail and pre-frail older adults: A systematic review and meta-analysis. Archives of Gerontology and Geriatrics. 2024;125(no pagination). | No specific results for the effect of protein |
| 1. Mills, S. R., et al. (2018). "Can fortified foods and snacks increase the energy and protein intake of hospitalised older patients? A systematic review." Journal of Human Nutrition & Dietetics 31(3): 379-389 | No results for outcomes of interest |
| 1. Morilla-Herrera, J., et al. (2016). "Effectiveness of food-based fortification in older people a systematic review and meta-analysis." Journal of Nutrition, Health & Aging 20(2): 178-184. | No results for outcomes of interest |
| 1. Dewansingh, P., et al. (2021). "Protein, Calcium, Vitamin D Intake and 25(OH)D Status in Normal Weight, Overweight, and Obese Older Adults: A Systematic Review and Meta-Analysis." Frontiers in Nutrition 8: 718658 | No results for outcomes of interest |
| 1. Coelho-Junior, H. J., et al. (2022). "Protein Intake and Sarcopenia in Older Adults: A Systematic Review and Meta-Analysis." International Journal of Environmental Research and Public Health 19(14) (no pagination). | No results for outcomes of interest |
| 1. Coelho-Junior, H. J., et al. (2022). "Protein Intake and Frailty in Older Adults: A Systematic Review and Meta-Analysis of Observational Studies." Nutrients 14(13): 2767-N.PAG. | No results for outcomes of interest |
| 1. Cawood, A. L., et al. (2011). "Systematic review and meta-analysis of the effects of high protein oral nutritional supplements on strength." Proceedings of the 7 | Conference abstract |
| 1. Goisser, S., et al. (2019). "Nutritional interventions for treating sarcopenia in older persons: A systematic review and meta-analysis project following the standards of the Cochrane Collaboration." European Geriatric Medicine 10(Supplement 1): S260-S261 | Conference abstract |
| 1. Hida, A., et al. (2017). "Effect of dietary and exercise interventions in sarcopenic, pre-frail and frail older adults." Annals of Nutrition and Metabolism 71(Supplement 2): 869 | Conference abstract |
| 1. Hettiarachchi, J., et al. (2021). "The effect of dose, frequency and timing of protein supplementation, on muscle mass in older adults by population: a systematic review and meta-analysis." Clinical Nutrition ESPEN 46: S739 | Conference abstract |
| 1. Martinez, A. C. (2019). "Proteins and sarcopenia." Annals of Nutrition and University Press on behalf of the American Society for Nutrition. | Conference abstract |
| 1. Saguez, R., et al. (2021). "Resistance exercise and nutritional supplementation in community-dwelling older people with sarcopenia: Systematic review and meta-analysis." European Geriatric Medicine 12(SUPPL 1): S369 | Conference abstract |
| 1. Verstappen, J., et al. (2018). "Impact of dietary protein supplementation on length of hospital stay and mortality in older adults: A systematic review and meta-analysis...European Society for Clinical Nutrition and Metabolism (ESPEN) 40th Congress, September 1-4, 2018, Madrid, Spain." Clinical Nutrition 37: S336-S336 | Conference abstract |
| 1. Nunes E, Colenso-Semple L, McKellar S, Yau T, Ali M, Fitzpatrick-Lewis D, et al. Increasing Protein Ingestion Minimally Increases Lean Mass and Muscle Strength in Subjects Enrolled in Resistance Exercise Training a Systematic Review and Meta-Analysis. Current Developments in Nutrition. 2022;6(Supplement 1):1169. | Conference abstract |
| 1. Bauer, J. M. and R. Diekmann (2015). "Protein supplementation with aging." Current Opinion in Clinical Nutrition & Metabolic Care 18(1): 24-31 | Reviews without a formal numerical synthesis |
| 1. Beaudart C, Dawson A, Shaw S, Harvey NC, Kanis J, Binkley N, et al. Nutrition and physical activity in the prevention and treatment of sarcopenia: systematic review. Osteoporosis International. 2017;28:1817-33. | Reviews without a formal numerical synthesis |
| 1. Camargo LDR, Doneda D, Oliveira VR. Whey protein ingestion in elderly diet and the association with physical, performance and clinical outcomes. Experimental Gerontology. 2020;137 (no pagination). | Reviews without a formal numerical synthesis |
| 1. Dorrington, N., et al. (2020). "A Review of Nutritional Requirements of Adults Aged >=65 Years in the UK." The Journal of nutrition. | Reviews without a formal numerical synthesis |
| 1. Lancha Jr, A. H., et al. (2017). "Dietary protein supplementation in the elderly for limiting muscle mass loss." Amino Acids 49(1): 33-47. | Reviews without a formal numerical synthesis |
| 1. Gade J, Pedersen RJ, Beck AM. Effect of Protein or Essential Amino Acid Supplementation During Prolonged Resistance Exercise Training in Older Adults on Body Composition, Muscle Strength, and Physical Performance Parameters: A Systematic Review. Rehabilitation Process & Outcome. 2018(7):1-. | Reviews without a formal numerical synthesis |
| 1. Nakanishi, N., et al. (2022). "Impact of Energy and Protein Delivery to Critically Ill Patients: A Systematic Review and Meta-Analysis of Randomized Controlled Trials." Nutrients 14(22) (no pagination). | Reviews without a formal numerical synthesis |
| 1. Nishimura, Y., et al. (2021). "Dietary protein requirements and recommendations for healthy older adults: a critical narrative review of the scientific evidence." Nutrition research reviews: 1-17. | Reviews without a formal numerical synthesis |
| 1. Nowson, C. and S. O’Connell (2015). "Protein requirements and recommendations for older people: a review." Nutrients 7(8): 6874-6899. | Reviews without a formal numerical synthesis |
| 1. Verdijk, L. B. (2021). "Nutritional supplementation to enhance the efficacy of exercise training in older adults: what is the evidence from the latest randomized controlled trials?" Current Opinion in Clinical Nutrition & Metabolic Care 24(6): 504-510. | Reviews without a formal numerical synthesis |
| 1. del Peral JAR, Sonia Gracia Josa M. Protein supplements in the treatment and prevention of sarcopenia. A systematic review. [Spanish]. Gerokomos. 2019;30(1):23-7. | Reviews without a formal numerical synthesis |
| 1. Grigg M, Arora M, Diwan AD. Role of nutritional supplementation in elderly patients with hip fractures. Journal of Orthopaedic Translation. 2014;2(1):26-34. | Reviews without a formal numerical synthesis |
| 1. Hengeveld LM, de Goede J, Afman LA, Bakker SJL, Beulens JWJ, Blaak EE, et al. Health Effects of Increasing Protein Intake Above the Current Population Reference Intake in Older Adults: A Systematic Review of the Health Council of the Netherlands. Advances in nutrition (Bethesda, Md). 2022;13(4):1083-117. | Reviews without a formal numerical synthesis |
| 1. Hou V, Madden K. Assessing the Effects of Dietary Protein Supplementation on Sarcopenia in Community-Dwelling Older Adults. Canadian Geriatrics Journal. 2022;25(4):390-403. | Reviews without a formal numerical synthesis |
| 1. Malafarina V, Uriz-Otano F, Iniesta R, Gil-Guerrero L. Effectiveness of Nutritional Supplementation on Muscle Mass in Treatment of Sarcopenia in Old Age: A Systematic Review. Journal of the American Medical Directors Association. 2013;14(1):10-7. | Reviews without a formal numerical synthesis |
| 1. Mareschal J, Genton L, Collet T-H, Graf C. Nutritional intervention to prevent the functional decline in community-dwelling older adults: a systematic review. Nutrients. 2020;12(9):2820. | Reviews without a formal numerical synthesis |
| 1. Naseeb MA, Volpe SL. Protein and exercise in the prevention of sarcopenia and aging. Nutrition research. 2017;40:1-20. | Reviews without a formal numerical synthesis |
| 1. Pedersen AN, Cederholm T. Health effects of protein intake in healthy elderly populations: a systematic literature review. Food & nutrition research. 2014;58(1):23364. | Reviews without a formal numerical synthesis |
| 1. Theodorakopoulos C, Jones J, Bannerman E, Greig CA. Effectiveness of nutritional and exercise interventions to improve body composition and muscle strength or function in sarcopenic obese older adults: a systematic review. Nutrition research. 2017;43:3-15. | Reviews without a formal numerical synthesis |
| 1. Thomas DK, Quinn MA, Saunders DH, Greig CA. Protein Supplementation Does Not Significantly Augment the Effects of Resistance Exercise Training in Older Adults: A Systematic Review. Journal of the American Medical Directors Association. 2016;17(10):959.e1-.e9. | Reviews without a formal numerical synthesis |
| 1. Coelho-Junior, H. J., et al. (2022). "Protein Intake and Sarcopenia in Older Adults: A Systematic Review and Meta-Analysis." International Journal of Environmental Research and Public Health 19(14) (no pagination). | No results from RCTs or nRCTs |
| 1. Coelho-Junior, H. J., et al. (2022). "Protein Intake and Frailty in Older Adults: A Systematic Review and Meta-Analysis of Observational Studies." Nutrients 14(13): 2767-N.PAG. | No results from RCTs or nRCTs |
| 1. Langsetmo, L., et al. (2020). "Low Protein Intake Irrespective of Source is Associated with Higher Mortality Among Older Community-Dwelling Men." Journal of Nutrition, Health & Aging 24(8): 900-905 | No results from RCTs or nRCTs |
| 1. Mendonca, N., et al. (2022). "Protein intake, physical activity and grip strength in European and North American community-dwelling older adults: a pooled analysis of individual participant data from four longitudinal ageing cohorts." British Journal of Nutrition. | No results from RCTs or nRCTs |
| 1. Mendonca, N., et al. (2021). "Low protein intake, physical activity, and physical function in European and North American community-dwelling older adults: a pooled analysis of four longitudinal aging cohorts." The American journal of clinical nutrition. | No results from RCTs or nRCTs |
| 1. Yaegashi A, Kimura T, Hirata T, Tamakoshi A. Association of dietary protein intake with skeletal muscle mass in older adults: A systematic review. Geriatrics & Gerontology International. 2021;21(12):1077-83. | No results from RCTs or nRCTs |
| 1. Mendonca NMP, Hengeveld LM, Presse N, Canhao H, Simonsick E, Kritchevsky SB, et al. Protein intake, physical activity and grip strength in European and North American community-dwelling older adults: A pooled analysis of individual participant data from four longitudinal ageing cohorts. British Journal of Nutrition. 2023;129(7):1221-31. | No results from RCTs or nRCTs |
| 1. Avenell, A. and H. H. G. Handoll (2003). "A systematic review of protein and energy supplementation for hip fracture aftercare in older people." European Journal of Clinical Nutrition 57(8): 895-903. | Review superseded by an update |
| 1. Vieira, A. F., et al. (2022). "Effects of Protein Supplementation Associated with Resistance Training on Body Composition and Muscle Strength in Older Adults: A Systematic Review of Systematic Reviews with Meta-analyses." Sports medicine (Auckland, N.Z.) 52(10): 2511-2522 | Umbrella Review |
